# The Revisions of the First Autobiography of AT Still, the Founder of Osteopathy, as a Step towards Integration in the American Healthcare System: A Comparative and Historiographic Review

**DOI:** 10.3390/healthcare12020130

**Published:** 2024-01-06

**Authors:** Silvia Clara Tuscano, Jason Haxton, Antonio Ciardo, Luigi Ciullo, Rafael Zegarra-Parodi

**Affiliations:** 1 Istituto Europeo per la Medicina Osteopatica (IEMO), 16122 Genova, Italy; silvia.tuscano@iemo.info (S.C.T.); luigi.ciullo@univerosteo.it (L.C.); 2 Museum of the Osteopathic Medicine, Kirksville, MO 63501, USA; jhaxton@atsu.edu; 3 Grupo Thuban-Fundación Europea de Medicina Tradicional Complementaria e Integrativa (FEMTCI), 28028 Madrid, Spain; aciardo@grupothuban.com; 4 Facultad de Ciencias de la Salud, Universidad Europea del Atlántico, 39011 Santander, Spain; 5 BMS Formation, 75116 Paris, France; 6 A.T. Still Research Institute, Kirksville, MO 63501, USA

**Keywords:** integrative care, osteopathy, osteopathic history, osteopathic medicine, Western health systems, professional identity

## Abstract

Introduction: Osteopathy was originally introduced in rural America in 1874 as a comprehensive therapeutic approach aimed at promoting health. This approach was distinct and often conflicting with conventional/allopathic therapeutic methods available at that time to fight disease. We argue that, in struggling to achieve recognition within the American healthcare system and within the educational academic field that was about to be structured, the American osteopathic profession tried to protect itself from the charges of sectarism by starting to embrace principles of the biomedical paradigm. Methods: A comparative and historiographic review of the second version of the autobiography of AT Still (1908), the founder of osteopathy, against the first (1897) was chosen as an example of the adaptation of the American osteopathic profession to its evolving academic environment. Results: Although there were only a few substantial variations, we argue that they aimed to dampen the non-biological components of osteopathy, namely, its philosophical, spiritual, religious, emotional, and Native American roots, in an effort to gain respect and recognition within the emerging gold standard of the Western medical system. The shift towards a distinct, fully integrated profession within regulated Western healthcare systems was perceived by many professionals as a threat to AT Still’s original ideas, and the trend started when he was alive. Conclusion: Our findings suggest that a crucial conversation regarding the future of the professional identity must take place within the osteopathic community.

## 1. Introduction

### 1.1. Osteopathy and Osteopathic Medicine Worldwide

According to the Osteopathic International Alliance, osteopathic healthcare is currently delivered in 46 countries worldwide by an estimated 200,000 clinicians, who can be distinguished in two groups of professionals. Firstly, osteopathic physicians in the USA, licensed to practice the full scope of medicine and surgery, provide osteopathic medicine. Secondly, outside the USA, osteopaths provide osteopathy with a hands-on scope of practice [1] All osteopathic professionals use a range of techniques, including “hands-on” manual techniques, for assessment and diagnosis to identify and then treat various conditions within a “person-centered” rather than “disease-centered” framework, and to promote health and well-being [1]. For example, in the United Kingdom, osteopaths are categorized as allied health professionals and face restrictions in advertising treatments, limited to those supported by evidence regarding the efficacy of their interventions, predominantly for musculoskeletal (MSK)-related conditions. Furthermore, they are obligated to refer patients in instances where there is limited or negative evidence supporting the effectiveness of manual therapy. Conversely, in France, the title of osteopathy is shared by both medical and non-medical healthcare professionals, as well as non-healthcare professionals. Their scope of practice is confined to the treatment of conditions based on manual palpatory findings associated with somatic dysfunctions, predominantly within an MSK-related framework. In other nations where osteopathic professionals lack statutory regulation, there exists the potential for them to assert a broader scope of practice extending beyond MSK-related conditions. This raises ethical concerns, as the absence of regulatory mechanisms may compromise the quality and standardization of care [2] but, despite different regulations and scopes of practices, most practitioners refer to the historical work of the first osteopaths when explaining the specific professional skills used in osteopathic care compared to other forms of manual therapy [1].

### 1.2. Osteopathy as a “Hands-On” Form of Alternative Medicine Seeking Recognition in the USA

Osteopathic education was established in rural America at the end of the 19th century by Andrew Taylor Still, who began his career as an orthodox Doctor of Medicine (MD) on the frontier in the 1850s. He later developed osteopathy and created the American School of Osteopathy (ASO) in 1892, thus becoming the first Doctor of Osteopathy (DO) and providing the impetus for the dissemination of this new discipline to many countries over the last century. Currently, he is referred to as Dr AT Still MD, DO within the osteopathic profession [3].

Osteopathy was originally established as a comprehensive therapeutic system and an alternative to the medical standards of that time [4]. Early osteopathic principles that guided patient care combined Western conventional and unconventional healthcare principles with traditional (Native American) healing principles, focusing on promoting health and not merely fighting disease [5,6]. Depending on the historical period and on the laws in force regulating healthcare across the world, the introduction of osteopathy as a new profession within the existing healthcare systems in various countries was usually followed by conflict as it sought acceptance and then recognition. This happened for several reasons: (i) standing up to the hostility of the established biomedical environment focusing on disease, and of other practitioners with similar “hands-on” scopes of practice; (ii) avoiding charges of sectarianism because of the promotion of health through natural/innate processes; and (iii) putting a stop to imitators who called themselves osteopaths in the absence of recognized academic institutions, thus discrediting the entire profession.

In the United States, the first professionals fought a long battle to be accepted within the USA’s health system. They originally called themselves “osteopaths” or “osteopathists” [7,8]; they later held the title of DO (Doctor of Osteopathy)—an American title not to be confused with the international non-USA acronym for Diplomate in Osteopathy; and, after 1940, they labeled themselves “osteopathic physicians” [9] (p. 110). In 1897, some ASO students founded the AAAO (American Association for the Advancement of Osteopathy), which, in 1901, became the AOA (American Osteopathic Association), and undertook the great endeavor of regulating, defining, and establishing the osteopathic profession. The AOA worked on two fronts: internally, trying to bridge the sometimes apparently irreconcilable conflicts between the practitioners about Dr AT Still MD, DO’s seminal perspectives on osteopathic principles; and, externally, striving to craft a cohesive and standardized profile for the profession, in close cooperation with the Associated Colleges of Osteopathy (ACO)—established in 1898 to develop the educational curriculum and create a list of recognized schools [10] (pp. 272–273). The AOA worked hard to promote osteopathy; for instance, in 1901, it launched the *Journal of the American Osteopathic Association*, and it also established standing committees on publication, legislation, and education [11] (p. 15), while, in 1902, some of its members became aware of the need to adopt a written Code of Ethics [12] (pp. 85–86).

### 1.3. The Need to Develop High Standards of Education to Secure the Future of This Emerging Profession

Besides stimulating research and disseminating original articles or case reports, the AOA hosted discussions and took decisions that determined the future of the profession in the USA, especially in the sectors of legal acknowledgement and education. The battles for legal recognition had to be fought in every single state, and sometimes new legislature could change a previously approved law, sending osteopathy back to square one. By 1901, osteopathy was recognized in 15 USA states, which meant that American DO graduates had to pass the exams of the medical boards—in some states, this meant that American DOs were examined (except for the questions concerning pharmacology) alongside medical doctors (MDs), in the existing medical boards, which occasionally admitted one American DO for representation. However, the AOA strived for the establishment of independent boards for osteopathic examination and registration, and, in 1913, seventeen of the thirty-nine states that had regulated osteopathy provided independent boards. This prompted the AOA to require the colleges to lengthen their curricula from 2 years to 3 years by 1904, even if this caused the closure of several schools [9] (p. 59).

Meanwhile, the American osteopathic community was torn by internal academic discussions, mainly regarding two important issues: (i) to determine the American DOs’ scope of practice, i.e., whether it should be equal to the full scope of medicine practice similar to MDs’ within the American healthcare system, or whether it should be focused only on “hands-on” medicine; and (ii) the subsequent educational material to be included in the curriculum, i.e., should pharmacology and the right to prescribe drugs be included or not? Osteopathy was indeed introduced as an exclusive “hands-on” alternative to allopathic medicine by Dr AT Still MD, DO, but many DOs wanted to expand their legal privileges and be allowed to practice surgery and obstetrics, and to use a limited range of drugs. They called themselves “broad osteopaths” and were opposed to the “lesion osteopaths”, who believed that the traditional idea developed by Dr AT Still MD, DO was better, and so they exclusively administered manual osteopathic treatments [3].

In the first decade of the 20th century, the USA standards of orthodox medical education were highly diverse: there were a few top-quality, university-affiliated colleges, and many profit-motivated proprietary schools. The AOA strived to maintain first-rate courses in all osteopathic educational institutions to protect the reputation of the profession and to ensure high-quality care for patients.

### 1.4. The Consequences of the Flexner Report on Medical and Osteopathic Education in the USA

However, both the AOA and the AMA were acutely conscious of the poor standards of their respective educational institutions. In 1904, the 160 MD-granting schools were surveyed and rated by the Council of Education of the American Medical Association (AMA), and only 82 were approved. Consequently, the Carnegie Foundation for the Advancement of Teaching and the American Medical Association (AMA) authorized Dr. Abraham Flexner, MD, to carry out a survey to rate 155 medical schools, the ones remaining after the previous survey. On the osteopathic side, the AOA Committee on Education appointed Dr ER Booth, DO, to carry out an inspection in all osteopathic colleges in 1903, in order to point out their strengths and weaknesses [9,10,13]. In 1910, a report by the same Committee highlighted the main problems of the schools, i.e., low entrance standards, poor basic science labs, a lack of sufficient clinical facilities, and inadequate teaching corps [9] (p. 90).

Flexner graduated as a teacher from Johns Hopkins University, which was established in 1876. He considered his alma mater an ideal model for medical education: this institution enforced a formal college education admission requirement primarily focused on basic sciences, and it featured a four-year medical curriculum that placed a remarkable emphasis on clinical and basic sciences. More specifically, Flexner stressed the importance of adhering to the scientific method and offering clinical training facilities. Furthermore, he recommended that adequately educated teachers were selected in sufficient numbers and well versed in research. An approved medical school should be affiliated with a university and provide “learning by doing” programs [14,15]. Flexner recommended that the number of medical schools be reduced to only 31 colleges, which would have to be consolidated as university schools, committed to research and academic excellence [16]. In his famous report (1910), Flexner also analyzed osteopathic schools, which he grouped in a separate chapter titled “Medical Sects”, together with the “homeopathists”, the “eclectics”, and the “physiomedicals”. According to Flexner, “The eight osteopathic schools fairly reek with commercialism. Their catalogues are a mass of hysterical exaggerations, alike of the earning and of the curative power of osteopathy” [9] (p. 163).

The AOA Board of Trustees greeted the Flexner Report with anger, maintaining that osteopathic colleges had performed well and demanding to be allowed to educate their students according to the profession’s needs. On the other hand, this might have been a formal response, since it did not match with the abovementioned 1910 report by AOA’s Committee on Education, which revealed shortcomings similar to those observed by Flexner [9] (pp. 89–90). In the wake of the Flexner report, many osteopathic schools took action to comply to the scientific approach within a biomedical paradigm, paralleled by the activity of the AOA committees and the Associated Colleges of Osteopathy, giving additional fuel to the internal debate about the scope of practice [3]. The Flexner report triggered the broad expansion of biomedical research in the few surviving medical schools adapting to the new gold standard of education, also propelled by large funding from the state and donations from benefactors. The biomedical model, which views disease as a result of physical or chemical abnormalities in the body, became the dominant model of medicine in the United States in the late 19th and early 20th centuries. This led to a new gold standard for medical education, which emphasized the study of science and technology. Medical schools began to require students to have a strong foundation in chemistry, physics, and biology, and to complete internships and residencies in hospitals [17,18]. This new gold standard had a significant impact on other forms of therapy, such as naturopathy, osteopathy, and homeopathy, which were not based on the biomedical model. Anything outside the new paradigm was labeled charlatanism—whether it was a “nonconformist” approach to medicine and psychiatry or pertained to natural/traditional theories, such as homeopathy, naturopathy, eclectic therapy, physical therapy, osteopathy, and chiropractic [19]. These therapies were often dismissed as “quackery” by conventional doctors, and their practitioners were denied access to hospitals and other medical resources.

The consequences of this marginalization were severe for American DOs. However, practitioners of alternative therapies also often had to operate outside the mainstream medical system, and their patients were often denied insurance coverage for their treatments. This made it difficult for these therapies to gain widespread acceptance and made it more difficult for people to access them. The marginalization of alternative therapies also had a negative impact on medical research. Because these therapies were not considered to be legitimate medicine, they were often excluded from clinical trials and other research studies. This made it difficult to assess their safety and efficacy, and it perpetuated the myth that they were ineffective.

### 1.5. The Crossroads for the Osteopathic Profession Regarding Dr AT Still MD, DO’s Original Ideas

Internal discussions on the scope of practice had already emerged in the osteopathic community before the turn of the century. In 1892, the ASO offered a short course, consisting of anatomy, philosophy of medicine, and clinical observation, but, in 1897, it started to incorporate classes on all basic sciences, minor surgery, and obstetrics [20], teaching students how to use anesthetics, antiseptics, and antidotes. According to Dr AT Still MD, DO, this was enough to satisfyingly train general practitioners. However, many American “broad osteopaths” felt that there was no reason to refuse any other therapeutic modalities that they considered to be of value and opposed the so-called “lesionists” or “lesion” osteopaths [9] (p. 69).

Both groups, however, seemed to agree about finding a common, scientific basis for their new science, in line with the new gold standard in medical education, i.e., the biomedical system as established by the Flexner report. Thus, they tried to wipe out any reference to controversial fields such as clairvoyance, spiritualism, native American culture, and other ideas that, in some ways, constituted some of the founding pillars of Dr AT Still MD, DO’s philosophy [21,22,23].

The Committee on Publication of the AOA oversaw the dissemination of osteopathic texts within the profession and promoted their distribution to the public. New osteopathic literature was welcome, deemed worthy, and advertised. For instance, in 1908 [24], the Committee on Legislation argued, “what kind of impression does it make when fighting to establish osteopathy as an independent system of practice, to have it shown from our school catalogues that we use in preparing practitioners for this independent system all and only medical texts?” Additionally, “Every practicing osteopath should have all our own books and the schools should at least give them a preference in their catalogues and require their study for graduation” [24] (p. 130). Furthermore, in 1908, the JAOA boasted that more than twenty-five osteopathic books had been published, mostly penned by the faculty of the ASO and of the other osteopathic institutions [25] (p. 181).

### 1.6. The Dr AT Still, MD, DO Autobiography Written in 1897

The first edition of Dr AT Still MD, DO’s autobiography was written in 1897 and published in March 1898, and it was sold for USD five. It was a sincere chronicle of the events and thoughts that had brought the founder to discover osteopathy in 1874 and to develop it into a new science of medicine. Dr AT Still MD, DO did not belong to the academic world. He was born into an affluent and educated pioneer family living on the frontier, where his father was a Methodist preacher and physician. From an early age, he perused major medical books, verifying firsthand in nature the information he gathered from texts. Following the conventions of the time, Dr AT Still MD, DO acquired the title of MD through apprenticeship under his father, who managed the Wakarusa Indian mission in 1852–1853, treating Indians suffering from erysipelas, fevers, so-called flux, pneumonia, and cholera [26] (p. 45). To prepare for his apprenticeship during which he also learned the Shawnee language and culture, Dr AT Still MD, DO purchased Robley Dunglison’s book *Practice of Medicine* [27] (p. 25). From the early to mid-1850s and spanning over two decades, he engaged in the practice of medicine in Kansas as a conventional physician. During this period, he continued his medical studies under the guidance of his father, but it is important to note that during his time in Kansas, there was no established system of medical licensure, and possessing a medical school diploma was neither a requirement nor an expectation for being recognized as qualified to practice medicine [8]. Dr AT Still MD, DO harbored growing doubts on orthodox medicine after losing three children to spinal meningitis in 1864, despite the attendance of four of the most learned MDs of the land. After discovering osteopathy on June 22, 1874 through a vision, he was ousted by the religious, social and medical community because of his unconventional ideas, transferred to Kirksville, Missouri, and started roaming the country as an itinerant bonesetter [26] (pp. 136–137). In the course of his relentless research efforts, Dr AT Still MD, DO became acquainted with “irregular” practitioners, labeled as such by mainstream medical professionals, who thrived in the unregulated American environment. Among the practitioners inclined to trust in more natural powers were the eclectic physicians offering a mix of botanic, Indian, and conservative medicine, as well as midwives, homeopaths, but also “hands-on therapists” representing new trends within orthodox medicine, such as the Ling System introduced by George H. Taylor, stemming from the medical gymnastics of ancient physicians [28].

As mentioned, the first version of Dr AT Still MD, DO’s autobiography was written in 1897. It is true that osteopathic textbooks might have been more urgent, but there was much curiosity from the public about the story of his life and his discovery of osteopathy [26] (p. 183). Dr AT Still MD, DO’s book was well disseminated, because (i) at the time, there were no other printed sources narrating his life; (ii) the autobiography contained various lectures and addresses that he gave in the 1890s; and (iii) he used to donate copies to ASO students [29]. Therefore, it was an appealing and attention-grabbing book for those who wanted to understand more about this new discipline. The autobiography was sold out after a few years and a reprint was impossible since the original plates were damaged in a fire in 1907. Thus, it was decided to release a second edition, revised with the help of Dr EB Veazie, DO, and Prof Bean, DO [30]. On the basis of some research, we speculate that Ella B. Veazie might have been a 1906 ASO graduate [31], who also authored several anatomical drawings [32], while Professor Bean could have been Arthur Sanders Bean, who graduated in 1904 [33], perhaps the same A.S. Bean mentioned in Booth’s *History of Osteopathy* as one of the osteopaths who contributed to the profession by writing books and periodicals [10], and who also penned an article in 1919 [34].

An important critical analysis on the content of Dr AT Still MD, DO’s autobiography was provided by Gevitz in 2014, describing Still’s evolution on how he described himself first as an MD, without earning a formal diploma, later as a magnetic healer and then as a bonesetter, before proposing a coherent new form of healing method he later named osteopathy [35]. In his paper, Gevitz outlines that Dr AT Still, MD, DO’s autobiography was published more than a century ago, at a time where there were no academic standards for such publications and acknowledge some frustration for current readers who will find a selected collection of anecdotal evidences that did not undergo our current peer-review processes that usually require references to support the content [35]. Dr AT Still MD, DO’s fascinating personality featured a profound knowledge of nature, coupled with deep philosophical insight, an unconventional and curious mind, a candid sense of awe for God’s creation, incredible knowledge of anatomy, remarkable bonesetting skills, familiarity with the Shawnee native Indians’ language and culture [6], and an unshakable hatred of “heroic” medicine—which made him more of a “guru” than a scientific researcher.

### 1.7. The Revisions of the Second Edition of Dr AT Still, MD, DO’s Autobiography Occurring in the Specific Context of the American Medical Regulation Process and the Search for Recognition of the American Osteopathic Profession

Our considerations about this manuscript are rooted in a specific timeframe, i.e., the decade from 1898 to 1908. We argue that the changes made to the second edition of Dr AT Still MD, DO’s autobiography mirror the change in the general sentiment towards medicine matured in the osteopathic community as the gold standard in medical education was being shaped. Although the Flexner report would not be published until 1910, the AOA regularly conducted inspections in the osteopathic colleges—as did the AMA—and issued guidelines on the basis of the results. For instance, the inspection carried out in 1903 was instrumental to the mandatory inauguration of a three-year, twenty-seven-month osteopathy course by 1904 [9] (p. 59), while, in 1906, the AOA inspector complained that too many medical notions were administered through lectures, especially in the colleges of Chicago, Boston, and Los Angeles [9] (p. 59). We speculate that both categories of American DOs operating in the early 20th century, regardless of whether they defined themselves as “lesion” or “broad” American DOs, felt compelled to put aside some of the philosophical and spiritual aspects that were at the core of Dr AT Still, MD, DO’s original principles for the sake of recognition. Osteopathy was indeed founded on the principles of holism, which emphasizes the interconnectedness of body, mind and spirit. However, as osteopathy became more integrated into mainstream medicine, there was a growing emphasis on scientific evidence and a de-emphasizing of emotions and spirituality. This led to the perception of emotions and spirituality as “quackery” within osteopathy. Therefore, the implicit choice to move away from osteopathy’s holistic roots in the educational environment began in the early years of the profession.

The aim of this paper is to describe the modifications made to the second version of Dr AT Still MD, DO’s autobiography compared to the first, and to discuss them in light of the mainstream biomedical ideas that started to be established as the gold standard for medical education at that period of time. We hypothesize that some aspects of Dr AT Still MD, DO’s early radical thoughts and personal philosophy that were deemed to be eccentric or unacceptable had to be softened in the process of a young profession seeking recognition.

## 2. Methods

This study draws a comparison between the two versions of Dr AT Still MD, DO’s autobiography, available in the public domain, digitized from the originals. In December 2020, we downloaded the PDF format of the 1897 and the 1908 editions of Dr AT Still MD, DO’s autobiography from an internet library [36], as follows:

Still A.T. “Autobiography of Andrew T. Still with a History of the Discovery and Development of the Science of Osteopathy”, published by the Author, Kirksville, MO, 1897 [37] and Still A.T. “Autobiography of Andrew T. Still with a History of the Discovery and Development of the Science of Osteopathy”. Illustrated—Revised Edition, published by the Author, Kirksville, MO, 1908 [30].

Regarding our research method, we conducted a comparative analysis [38] of the two above-described versions of the book on a chapter-by-chapter basis within a historiography perspective, defined as “the writing of history based on the critical examination of sources, the selection of particular details from the authentic materials in those sources, and the synthesis of those details into a narrative that stands the test of critical examination” [39]. However, we did not limit our work to the description of differences but tried to extract insights about causal relationships within the framework of what May defined as a theory-development view [40] (p. 157) and what Tilly called the universalizing comparison [41] (p. 97). We construed the modifications made to Dr AT Still, MD, DO’s autobiography in light of the new, science-oriented medical paradigm arising in the USA at the beginning of the 20th century. This paradigm might have shaped the evolution of this relatively young profession, which was then seeking recognition, and might have led to the removal of professional skills and/or alternative concepts not acceptable by the dominant biomedical model in Western healthcare in order to survive.

Two versions of each chapter—from the 1897 and 1908 editions, respectively—were created and compared using the Draftable online software (Vesparum Capital Pty Ltd., Melbourne, Victoria, Australia, accessed on https://www.draftable.com/ or https://www.vesparum.com/ (accessed on 1 December 2023)). One author (S.C.T.) performed the whole task and a double check was later performed on random chapters by the coauthors, approximately involving 10% of the material.

### Assessment and Analysis

Each chapter of the 1908 edition was annotated on an Apple MacBook Air Retina 3-inch 2018 computer (Apple Computer, Inc, Cupertino, CA), using the built-in Apple Preview software (Apple Computer, version 10.0, Inc, Cupertino, CA). Formatting and layout differences were disregarded, and the following color codes were used:

- Yellow highlights indicated added text;
- Pink highlights indicated differences, and the original 1987 text was reported in the notes;
- Green highlights indicated all occurrences deemed worthy of further consideration or subsequent thought—sometimes, whole sentences were highlighted in green, while, in other cases, only a little green was added to yellow- and/or pink-highlighted sentences, to indicate minor importance.

The annotated chapters are available in the Supplementary Materials.

## 3. Results

On the whole, the findings have been classified into four categories to facilitate the comparison:

1. Grammar and syntactic text changes made by the reviewers to correct dates, measurements, and style and to improve readability;
2. Text changes introducing different nuances compared to the first edition;
3. Substantial changes to the text;
4. Pictures.

Whenever specific pages of Dr AT Still MD, DO’s autobiography are indicated in this paper, they always refer to the 1908 edition, i.e., the second version of the book. The differences in the reported quotations of the two versions of the book are italicized and underlined.

1. Grammar and syntactic text changes made by the reviewers to correct dates, measurements, and style and improve readability: These constitute the majority of the alterations, and almost each page features many of them. The reviewers also softened overly dogmatic statements and rectified dimensions. However, most of the editing consists of modifications and additions of new words in order to improve the readability or correct the syntax; the complete list of alterations can be found in the Supplementary Materials. To provide further insight, some examples are reported in Table 1.
2. Changes introducing different nuances to Dr AT Still MD, DO’s ideas and/or personality: These are not as numerous as those in the first category; however, they represent a large amount overall. In general, most amendments were made with the following aims:
  a. To soften overly harsh affirmations about the war—for instance, on pages 73–76, “we had the satisfaction of tearing down many flags” was changed to “we took down many flags”.
  b. To emphasize that Dr AT Still MD, DO was commissioned “major” (p. 75).
  c. To purge the word “grave-robber”, which Dr AT Still MD, DO had used in the first edition (pp. 84–85).
  d. To delete some playful wordings. As an example, see p. 112, where an “amusing scientific incident” was replaced with an “incident” in the second edition, and the subsequent text was also heavily amended and extended, emphasizing Dr AT Still MD, DO’s aversion to alcohol (see also the addition of “whisky” on page 170).
  e. To erase the referral to Indian healing methods by eliminating the following sentence on page 113: “I had no object in view when I pow-wowed the old gentleman, punched and twisted his abdomen, and told him of the awful ending of the sot, except a little street fun”.
  f. To remove Dr AT Still MD, DO’s reminiscence about considering to take his own life, by removing the following sentence: “I have long thought I might at some time be called to stop my useless life of misery and hours of lamentations” (p. 119). The subsequent paragraphs were also edited so that they could convey the impression of referring to a vision instead of a chronicle. On this page, Dr AT Still MD, DO writes about being saved from dejectedness by the news that his 10-year-old son had found a job to support the family. The 1897 original reads “With trembling gait my wife came to my side and said: ‘Look at our little boy of ten summers…”, while the same paragraph of the 1908 starts with “In a vision of the night of despair, I saw my wife who came to my side and said: ‘Look at our little boy of ten summers…”. Moreover, the subsequent sentences were changed accordingly, replacing “I listened” with “I seemed to listen” and “I saw” with “I seemed to see”.
  g. To stress Dr AT Still MD, DO’s good opinion of women (on p. 72, “wrote the golden words: ‘Forever free, without regard to race or color” was modified with an addition, “wrote the golden words: ‘Forever free, without regard to race or color,’ I will add—or sex”, and again on page 162). The cruel treatment that women had to endure because surgeons were ignorant about the law of parturition was also emphasized through the addition of the following sentence to page 133 of the 1908 edition: (Osteopathy] … teaches that lacerations to the mother and injury to the child by forceps are not necessary except in extreme cases of bone deformities”*)*.
  h. To limit and/or homogenize the spiritual/religious realm, as in the examples reported in Table 2.

The following few examples are also reported to point out some changes in the passages concerning the science of osteopathy and/or medicine [see Table 3].

3. Substantial changes

Substantial changes to the text are very few; in particular, they consist of the following:

- The addition of several songs, rhymes, and contributions to osteopathy, which were produced in the time interval following the first edition (after 1897) and preceding the second one (1908);
- The addition of an entirely new chapter (Chapter XXXIV, pp. 387–403);
- A sort of *damnatio memoriae* against Dr William Smith, who had held the first course of anatomy in 1892 and had contributed significantly to the development of the school—his name was removed in all instances in which it was feasible;
- The conversion into the masculine form of the cosmogony on pages 313–314, which originally described the Sun, the Moon, and all the planets in the feminine form (see Table 4);
- The removal of the first eight lines of Chapter XXIII, referring to a picture named *"Muscular System of Man"*, removed from the second edition.

4. Pictures

Most of the drawings and pictures remain the same, and the few—albeit important—changes are listed in Table 5 below.

**Figure 1:**
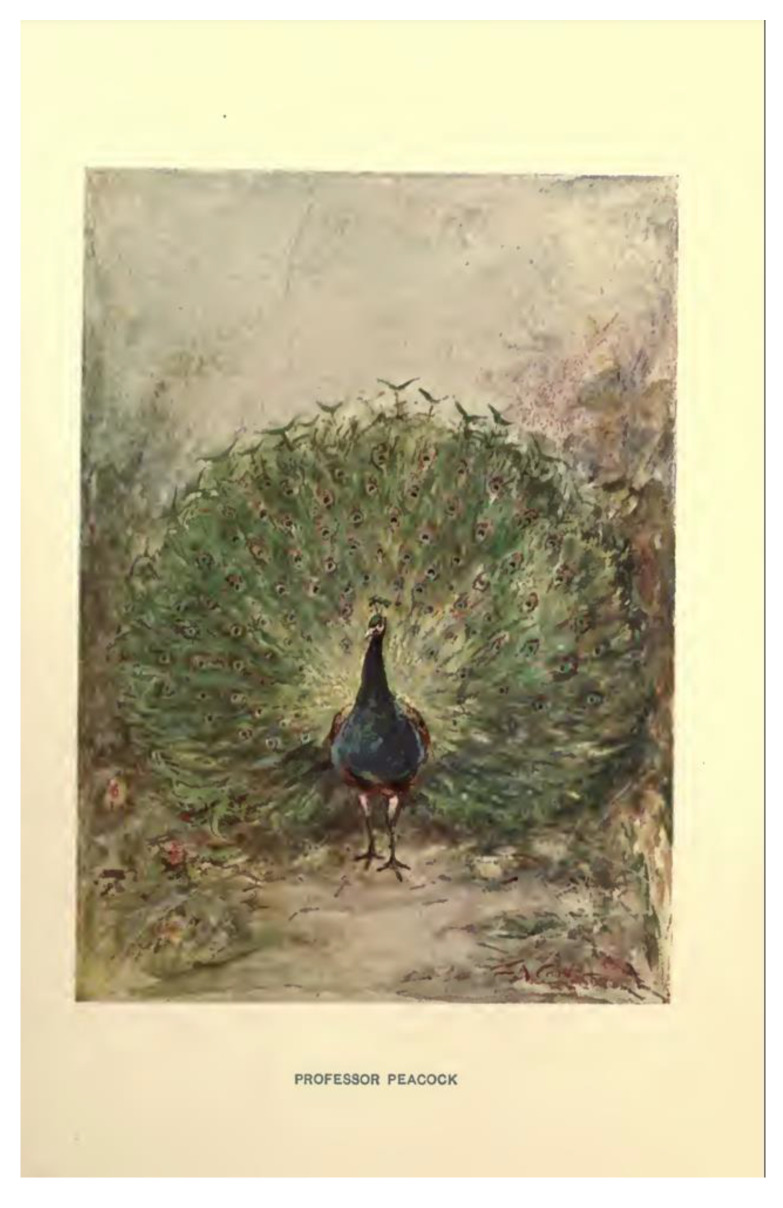
Picture *Professor Peacock* [37] (table between pages 316 and 317).

**Figure 2:**
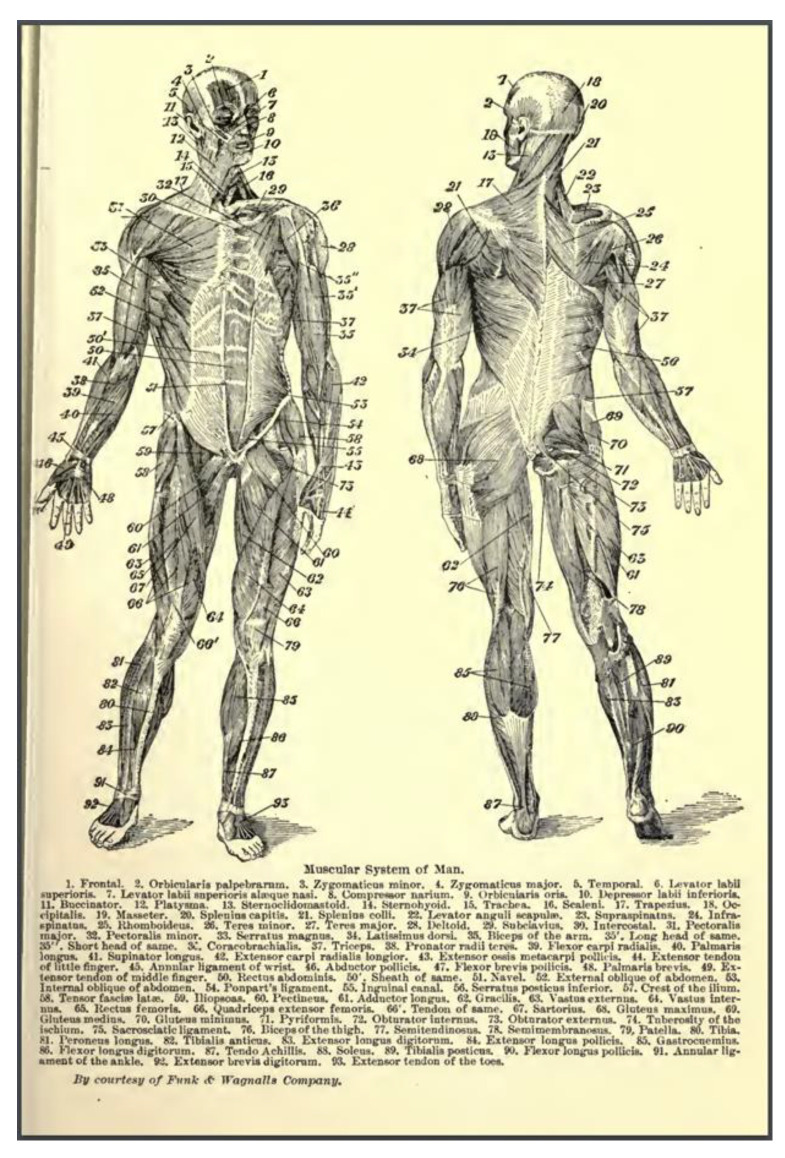
Picture *Muscular System of Man* [37] (table between pages 442 and 443).

In particular, the following might be interesting to note:

- Removal of the picture featuring the personification of the diseases;
- Removal of the portrait of Mrs. Anne Morris, who had typed the manuscript of the first edition;
- Removal of the “Professor Peacock” color picture (Figure 1);
- Removal of the table featuring the “Muscular System of Man” (Figure 2).

All the changes to the pictures are listed in Table 5.

## 4. Discussion

The purpose of this review was to carefully check the revisions of the second edition of Dr AT Still, MD, DO’s autobiography [5] and discuss the possible influence of the historical context of the changing medical educational environment in the USA as it adopted the biomedical paradigm. The fundamental principles of Dr AT Still, MD, DO’s initial theories to promote health focused not only on physical health but also on the spiritual and emotional components of each person as a unique individual. These non-physical aspects of health, which could not be biologically measured or scientifically evaluated, caused a professional identity problem for the osteopathic profession. The comparison of the original and the revised versions of Dr AT Still, MD, DO’s autobiography provides important insights into how professionals involved in the political an educational field handled the discussions around tradition and innovation to better adjust to the evolving healthcare environment [42].

### 4.1. Revisions Related to Non-Physical Components of Health, Disease, and Subsequent Care

Important modifications were introduced and were all related to the removal of potential threats within the specific American medical educational context as the field was about to establish the biomedical model as the gold standard. Other healthcare disciplines, such as osteopathy, which formally included non-biological components of health in its principles, were challenged and this led to an internal discussion among professionals when seeking respect and recognition.

#### 4.1.1. Less Use of Metaphors

**Figure 3:**
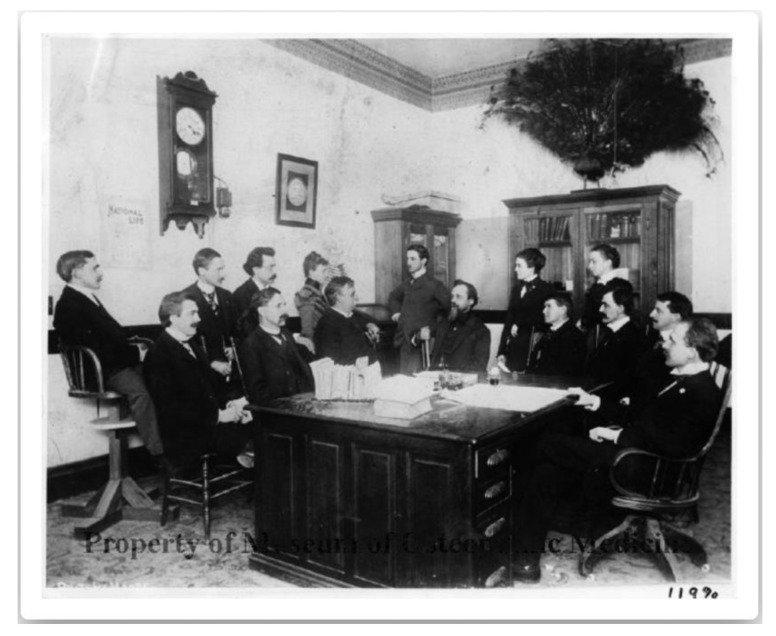
Picture *Andrew Taylor Still & ‘Official’ Family* about 1898 [44]—reprinted with permission from the Museum of Osteopathic Medicine, Kirksville, USA.

This might be testified by the removal of some pictures, e.g., *My name is Scarlet Fever; I live on little blue-eyed, fair-skinned children* between pages 160 and 161 of the 1897 edition, which portrayed the personification of disease in the guise of evil-looking men, and that of *Professor Peacock*, which was between pages 316 and 317 of the first version. The latter picture emphasized Dr AT Still MD, DO’s love of nature, which he referred to as a remarkable teacher. For example, as reported by Dr Arthur Hildreth, DO, during the second class at the ASO (1892–1893), Dr AT Still MD, DO interrupted a lecture about eczema, asking the students to wait: he returned home to fetch a stuffed duck and used the bird to point out its perfection and beauty, which could only be maintained by the correct function of the nerves and circulation [43] (pp. 42–43). He often mentioned “the great book of nature” and used to collect stuffed animals [23] (p. 181), [26] (p. 56). He likely found that peacocks were a great example of this concept, and one of these stuffed birds can be seen perched on top of a bookcase in a photograph taken at the ASO around May 1899 [44] (Figure 3).

#### 4.1.2. Fewer References to Non-Western Sociocultural Belief Systems towards Health

This could be evidenced by the modification in the feminine cosmology, which was reverted to the masculine in the revised edition of Dr AT Still MD, DO’s autobiography. It is very likely that the description of Mother Sun derives from the myths of the Shawnee Indians, who influenced the philosophy of the young Andrew Taylor while he stayed at the Wakarusa Mission to complete his medical apprenticeship with his father at the beginning of the 1850s [26].

In fact, several authors pointed out the Shawnee’s shift to a female Supreme Creator—for example, Voegelin: “comparative work shows the Shawnee to be unique among all the Eastern Woodlands Algonquinian-speaking peoples in possessing a female supreme deity and creator” [45]; Ethridge et al.: “The Shawnees are the only Algonquin tribe who believe they came from across the sea and that they were created by a woman who they call Co-cum-tha or Kokumthena, which means ‘grandmother’” [46]; and Lucas: “Many Native American tribal groups generally ascribed the masculine gender to the Great Spirit. The Shawnee, however, particularly before 1830 and the Indian Removal Act, held to a belief that the Supreme Creator was female” [47].

Furthermore, according to Niethammer, “The Shawnee […] believed that a deity they called ‘Our Grandmother’ was the creator of the universe and everything. As the supreme goddess, her creations were always beneficial to the Shawnee directly and to mankind in general. Our grandmother, who looked like an old woman with grey hair, as mentioned in every religious rite, and the large annual ceremonials were performed specifically to worship her, thereby preserving mankind and the world” [48].

Another author placed this change within a specific timeframe, approximately two decades before Dr AT Still MD, DO began the Wakarusa mission: “It is hypothesized that a shift from a warring way of life in the Ohio valley in the 1700s until their defeat in the war of 1812, to an agrarian life in Kansas (1830–1860s)—coupled with changes in tribal political structure, adjudication of persons accused of serious crimes, and social norms on marriage—altogether may well represent a sufficient background against which to see the Shawnee religion’s shift in devotional emphasis from the male Great Spirit to Our Grandmother, Kokomthena, a shift which has hitherto been seen to have taken place sometime between 1824 and the early 1930s” [49].

The integration of some aspects of the Shawnee’s traditional medicine into osteopathy has been pointed out by many authors [23,26,27,50], as also emphasized by various papers in the recent osteopathic literature [5,6,51,52].

### 4.2. Remaining Distinct in a Regulated Environment: Are Early Choices Still at Play in the Current Osteopathic Educational and Political Fields?

At the beginning of the last century, all colleges provided classes on osteopathic principles and practices (OPP) and taught the original ideas developed by Dr AT Still MD, DO, who stressed the importance of body, mind, and spirit, three key driving forces in human health and self-healing. Part of the profession was well aware of the spirit-based healing wisdom inherited from traditional medicine, but could not defend it within the growing biomedical paradigm [5]. Early osteopathy was attacked by physicians, who ridiculed its claims to be a cure-all and distrusted its manipulative approach—so distant from the well-learned physicians who did not touch their patients, and so close to the uneducated bonesetters who maintained that they did not need to study anatomy because of their “gift”.

Due to their vulnerability to accusations of sectarianism and cultism and the significant risk of having their licenses to practice revoked, American DOs made the decision to protect themselves by embracing the biomedical paradigm and the emerging dominant scientific mindset. The difficult issue of justifying their profession in a medical setting that preferred a more reductionist approach forced early American DOs to adapt their practice to accommodate the changing nature of healthcare. The nascent osteopathic profession was confronted with a pivotal decision: whether to adjust and remove certain foundational principles established by Dr AT Still MD, DO or to adhere steadfastly to his original work, even if it meant remaining outside the mainstream medical establishment. The evolution of osteopathic education in the United States was intrinsically linked to this choice [53,54]. The profession faced external pressures and internal debates regarding the scope of osteopathic practice and the content of the curriculum. Some advocated for a broader scope, seeking recognition within the healthcare system, while others insisted on preserving the traditional osteopathic principles.

Amid the radical transformation of the health sector, the members of the AOA’s Board of Trustees navigated the profession with intellectual humility [55,56], coping with the uncertainty and making the best choices for the survival of osteopathy. In the first three decades of the 20th century, orthodox medicine was unrecognizable compared to the “heroic” drug system of the 1850s. When the AOA allowed colleges to teach pharmacology in 1929, clinging to the traditional approach appeared anachronistic [20]. Osteopathic leaders chose the lesser evil in the short term, albeit possibly not in the best interest of the profession in the long term.

Furthermore, the most common health-threatening issues affecting patients in Dr AT Still MD, DO’s era dramatically altered over the course of the 20th century: the increasingly aging population of the Western world now suffered from chronic pain, obesity, and cardiovascular diseases. The existence of antibiotics, vaccinations, and surgery made compliance with early osteopathic principles unthinkable for many practitioners [57].

History repeated itself every time that osteopathy was introduced into a new country, triggering broader discussions within the profession about aligning with the biomedical model or staying true to the original ideas of Dr AT Still MD, DO [53,54]. The debate is so open that, although osteopathy is recognized in many countries, in others, it is at risk of being considered a pseudoscience [58]. This is confirmed by the existence of at least three types of osteopathy in the present day. The first is osteopathic medicine in the USA, where its development was paralleled by the continuous decline of hands-on treatment administration. According to a 2020 survey, more than 77% of American osteopathic physicians reported using manipulative treatments in less than 5% of their patients, while 56% reported not using them on any patient. Among the limiting factors, they mentioned a lack of time, lack of reimbursement, lack of institutional/practice support, and lack of confidence/proficiency [59]. The second is osteopathy as recognized by most countries issuing a regulation, where it is a type of physiotherapy (as, for instance, in the UK and in France). Often, the consistent efforts of the professional orders, which aimed to achieve better compliance with the evidence-based medicine (EBM) paradigm—a concept introduced 100 years after the first educational institute in osteopathy was created—result in substantial limitations to osteopathy’s scope of practice. The third is unregulated osteopathy in countries where the profession exists in a sort of limbo—lacking a legal framework and homogeneous education standards and having no way to ban imitators.

### 4.3. Different Sociocultural Contexts to Interpret Historical Osteopathic Principles

In the 20th century, the trend of moving away from osteopathy’s holistic foundation persisted. A standardized curriculum for osteopathic medical schools was developed in 1914 by the American Osteopathic Association (AOA). With little emphasis on the mind–body link or spirituality, this program placed a strong emphasis on biological science and clinical competencies. However, such concepts are also at the core of the alternative medicines that have gained more and more acceptance in recent years [2]. Today, many people use these treatments either in addition to or instead of traditional therapy. This is a result of a combination of factors, including the rising costs of healthcare, the growing unhappiness with the negative side effects of traditional pharmaceuticals, and the increased acceptance of holistic approaches to health and wellbeing.

Osteopathic education must make a crucial decision: either it changes/removes part of the basic content or it adheres to Dr AT Still MD, DO’s ideas, remaining outside of the mainstream medical establishment. Both strategies have advantages and disadvantages. Osteopathic education might be brought more in line with conventional medicine and made more appealing to students and businesses by changing or eliminating the early curriculum. Furthermore, it may be claimed that parts of the traditional osteopathic doctrines are no longer valid and backed by research. However, doing so would also entail departing from osteopathy’s fundamental tenets [60]. It might be possible to maintain the integrity of osteopathic education by adhering to Dr AT Still MD, DO’s ideas, but doing so could make it more challenging for osteopathic doctors to compete with allopathic practitioners for employment. A decolonial perspective, i.e., moving away from Eurocentric ideologies that imposed superiority and privilege over Indigenous communities, has recently been proposed and may guide further discussions towards non-physical components of health that were introduced at the beginning of the osteopathic profession [6], inherited from traditional (Native American) culture and reintroduced in 2002 by the American osteopathic profession [61]. The core tenets of Native American healing traditions revolve around the spiritual dimension, emphasizing the non-physical reality of patients as a conducive space for healing. According to this viewpoint, all diseases originate and conclude within the individual’s spirit. Ceremonies and rituals, varying across tribes but grounded in common principles, are utilized to achieve wholeness through a holistic approach that considers the body, mind, emotions, and spirit. This holistic perspective extends to the interconnectedness of all living things, encompassing people, nature, spirits, and the life force. In the pursuit of restoring health, the primary focus and treatment considerations are directed towards the ‘immortal soul’, symbolically positioned at the center of the sacred Medicine Wheel. This placement aims to foster balance among the components of the body, mind, spirit, and emotions in each quadrant [52]. In fact, even if many American osteopathic practitioners started to embrace the biomedical paradigm to gain respect and recognition from mainstream medicine since its early stages, and since there is no explicit acknowledgment of non-physical health’s components within its foundational principles, it may still be contended that osteopathy’s exclusive emphasis on the body–mind–spirit dynamic interaction is its most valuable feature [62].

Finally, according to our view, the osteopathic profession developed along different pathways because its leaders had a high degree of intellectual humility, characterized by openness, tolerance of ambiguity, and low dogmatism [55]. The internal debate among osteopathic practitioners was not based on unfounded confidence in individual leaders’ beliefs, but on the endeavor to seek the best solution for the unfolding of osteopathy in the midst of vastly differing circumstances as compared with the initial background conditions of Dr AT Still MD, DO’s time [56]. Leaders in the osteopathic educational field made different choices in different countries because they were eager to obtain and evaluate the information at hand; thus, their decisions were not dogmatic but specifically conceived to adjust to different healthcare environments—they had to make decisions to the best of their ability, given the available working knowledge within the scope of applicable laws in the health system of each particular country.

### 4.4. Current Perspectives for Osteopathic Education More than a Century after Dr. A.T. Still MD, DO’s Autobiography and the Flexner Report

Osteopathic education was strongly influenced by the Flexner Report on medical education, after which early academics established models for practice and education complying to scientific medical approaches focused on the specific clinical effects of manual therapy. Such patient-centered models are now challenged by current evidence and there is a trend in osteopathic education and practice to establish person-centered models [63] to better fit with modern patients’ values and expectations. Indeed, an aging society, the predominance of chronic versus acute illnesses, comorbidities and sedentary lifestyles, among others, are factors that explain why modern patients’ needs differ from those when Dr AT Still, MD, DO established osteopathy [64].

For example, recent studies have highlighted that patients’ experiences of pain do not rely solely on the understanding of the biology but rather entail intricate neural processes, encompassing sensory, emotional, cognitive, and interoceptive components specific to each person. Therefore, the study of contextual factors has been introduced in manual therapies to better fit patients’ values and expectations, as these factors can affect clinical outcomes [65]. The current focus in clinical care extends beyond the application of manual procedures to encompass multidimensional facets of the therapeutic encounter. This involves the practitioner, the patient, the patient–provider relationship and setting. These elements have the potential to evoke not only biological but also psychological responses, ultimately influencing clinical outcomes through placebo and nocebo effects, either positively or negatively. Patients’ expectations regarding treatment, the verbal suggestions provided by the physical therapist, and the strengthened therapeutic alliance between the patient and the physical therapist have all been documented to enhance outcomes in various domains, including pain, disability, expectation, and satisfaction [66]. This contemporary, humanistic, and holistic perspective within the field of health sciences emphasizes that practitioners should address the biological, psychological, and social dimensions of illness concurrently, aiming to enhance their understanding and responsiveness to patients’ suffering.

This biopsychosocial model of disease, introduced in recent decades, acknowledges the profound interconnectedness of the biological, psychological, and social dimensions in humans, which seem to align with the seminal ideas expressed by Dr. AT Still MD, DO in his autobiography. The enhancement in humanization in medicine and the empowerment of patients have consistently progressed through the incorporation of the patient’s subjective experience, the valuation of the patient–clinician relationship, and the assignment of new roles to the patient in clinical decision making [67]. Further, there is a growing consensus among researchers that the biopsychosocial model should be broadened to incorporate the spiritual dimension in healthcare [68] and this could be promoted by osteopathic practitioners [69]. Although few studies have examined the specific influence of the spiritual dimension in healthcare on the experience of pain, we argue that a biopsychosocial–spiritual approach would be in line with the early osteopathic principles stated by Dr. AT Still MD, DO. Indeed, a potential shift back to the traditional approach, aligning with the early principles articulated by Dr AT Still MD, DO as discussed in our manuscript, could help in redefining the scope of practice of the osteopathic profession in the manual therapy field. Osteopathic care ought not to confine itself solely to the musculoskeletal perspective but rather return to its initial whole-person approach that promotes health.

### 4.5. Limitations

The comparison between the two versions of Dr AT Still MD, DO’s autobiography was performed manually; so, it was prone to human mistakes in highlighting the differences.

After highlighting the changes, we pragmatically created a historical narrative of contingencies and interpreted the changes that we observed to validate our point of view, i.e., that the new, science-oriented medical paradigm rising in the USA at the beginning of the 20th century might have shaped the evolution of the osteopathic profession. This, of course, poses limitations to the historical interpretation of our study in this specific context [40].

## 5. Conclusions

We argue that 150 years after its conception in the mind of Dr AT Still MD, DO in 1874 and the creation of the first educational institution in 1892, some of the early osteopathic tenets and non-physical components of health, such as the spiritual/existential dimension, seem to have been modified or put aside in academic training to fit the needs of a changing healthcare sector that established the primacy of the biological component of health. This process started early, as shown in our comparison of the revised version of his autobiography when he was still alive.

Osteopathy has evolved considerably throughout the years, despite its lengthy history and distinctive philosophical foundations. Compared to Dr AT Still MD, DO’s initial idea, the reputation of osteopathic care worldwide is probably mostly due to positive clinical outcomes experienced by patients, and this profession is now more widely accepted in conventional medical systems. While there is little question that this integration has improved the quality of osteopathic care, it has also weakened some of the crucial insights that gave rise to osteopathy, such as its focus on the importance of the spiritual and emotional dimensions in the healing process. As a consequence, many osteopathic practitioners struggle between their commitment to Dr AT Still MD, DO’s original vision and the needs of a contemporary healthcare system that frequently favors speed and efficiency above holistic treatment.

We believe that a crucial conversation regarding the profession’s future must take place within the osteopathic community. By being aware of the specific historical context of the origin of osteopathic education in the USA, we may start to rethink how Dr AT Still MD, DO’s seminal concepts can be integrated into current modern care in a way that is both appropriate to the requirements of person-centered care and informed whole-person management of health, disease and illness. By doing this, we can ensure that the osteopathic profession continues to deserve its popularity among patients and remain a strong and influential force in healthcare.

## Figures and Tables

**Table 1:** A few examples of grammar and syntactic text changes.

Page (1908)	1897	1908
17	I suppose I *bawled*, and filled the bill of nature in the baby life. My mother was as others who had five or six *angels* to yell all night for her comfort.	I suppose I *cried*, and filled the bill of nature in the baby life. My mother was as others who had five or six *children* to yell all night for her comfort.
47	He raised his head two feet *in the air, and fixed those basilisk orbs* on me.	He raised his head two feet *above the ground, and fixed his eyes* on me.
58	This feeling of duty to free all and let each person have an equal chance to so live this life as a part of a vast eternity, preparatory to *joys immortal, which were bought and paid for by the life and blood of the Son of God*, continued to grow…	This feeling of duty to free all and let each person have an equal chance to so live this life as a part of a vast eternity, preparatory to *another life*, continued to grow…
96	I determined to try my luck *with what I then thought to be a new discovery*.	I determined to try my luck *in the introduction of what I had proven to be a new discovery and a remedy for human ills.*
101	A few months later *I found a man in great distress with asthma. I got off my horse and “hoodledooed”* him.	A few months later *as I was driving across the country on business, I found a man in great distress, suffering with an attack of asthma. The day was cold but the man sat out of doors astride a chair with his face to the back of it; he was gasping for breath and suffering so much that his family, helpless to relieve him, stood around him crying.* *I quickly dismounted and “hoodledooed” him, or in other words, I treated him, giving him relief at once, and he has had no return of the asthma during the six years which have passed since the treatment was given* him.
193	…when driven by the power of *life at the command of God, who gives power to all elements of force that exist beneath the great throne of mind*…	…when driven by the power of *life, which controls all the elements of force that exist*…
232	Osteopathy—a drugless science—finds the utero- genital nerves *made tight by the fastening of certain segments*.	Osteopathy—a drugless science—finds the utero- genital nerves *deranged by irritation*.

**Table 2:** A few examples of changes in the spiritual/religious realm.

Page (1908)	1897	1908
163	We are not enrolled under the banner of a *theologian.*	We are not enrolled under the banner of a *theorist*.
178	…I have been visited by *the visions of* the night…	…I have been visited by *visions in* the night…
185	*God* would not be forgetful […] and there is much evidence that *mind* is imparted to the corpuscles of the blood…	*Nature* would not be forgetful […] and there is much evidence that *knowledge* is imparted to the corpuscles of the blood …
186	You dare not assert that *the Deity*…	You dare not assert that *God*…
202	Death is *completed work of development of the sum total of effect to a finished work of nature.*	Death is the *end or the sum total of effects*.
208	… angels and worlds, are atoms *of which you are composed*. […] Therefore be kind in thought to the atoms of life, *or in death you will be borne to the grave by the beasts of burden who carry nothing to the tombs but the bodies of heedless stupidity, the mourners being the asses who cry and bray over the loss of their dear brother.*	… angels and worlds, are atoms. […] Therefore be kind in thought to the atoms of life.
209	Let us reason with a *faith* that nature does know…	Let us reason with a *thought* that nature does know…
291	…by simply adjusting the vocal *organs. Deity created the organs, and also the law of their adjustment when out of order; neither did He mistake in the creation,* nor in the law. … produced by *the use of calomel alone*.	…by simply adjusting the vocal *structure. Nature formed the organs, and framed the law of their adjustment and made no mistake in the formation*, nor in the law… produced by *them.*
330	He is surprised to find that man *is made by the eternal,* unerring Architect.	He is surprised to find that man *was made by an* unerring Architect.
330	The *thoughts of God himself are* found in every drop of your blood.	The *wisdom of Nature’s architect is* found in every drop of your blood.
332	…from the bosom of *God*.	…from the bosom of *Nature*.
343	The arteries bring the blood *and wash it with the spirit of life.*	The arteries bring the blood *of life and construct man, beast and all other bodies.*

**Table 3:** A few examples of changes concerning osteopathy or medicine.

Page (1908)	1897	1908
96	I determined to try my luck *with what I then thought to be a new discovery*.	I determined to try my luck *in the introduction of what I had proven to be a new discovery and a remedy for human ills.*
133	They know it falls to their lot to bear all the suffering and lacerations; therefore it is reasonable to suppose, for the sake of their sex, they will continue *the study of the laws* of parturition *to* a comprehensive and practical knowledge of all the principles belonging to this branch of Osteopathy.	They know it falls to their lot to bear all the suffering and lacerations *received through the ignorance of the doctor*; therefore it is reasonable to suppose, for the sake of their sex, they will continue *to study the law* of parturition *and gain* a comprehensive and practical knowledge of all the principles belonging to this branch of Osteopathy, *which teaches that lacerations to the mother and injury to the child by forceps are not necessary except in extreme cases of bone deformities*.
159	…that arterial action has been increased by heat to such velocity that veins cannot return *blood. Contract veins, and stop* the equality of exchange between veins and arteries.	…that arterial action has been increased by *sun*-heat to such velocity that veins cannot return *blood normally, but they become contracted, stopping* the equality of exchange between veins and arteries. *Then a chill follows for a short time, then fever*.
182	He who wished to successfully solve the problem of disease or *deformities* of any *kinds in all cases* without exception would find one or more *obstruction* in some artery, *or some of its branches*.	He who wished to successfully solve the problem of disease or *deformity* of any *kind in every case* without exception would find one or more *obstructions* in some artery, *or vein*.
182	…further proclaimed that the *brain* of man was God’s drug-store…	…further proclaimed that the *body* of man was God’s drug-store…
184	Greek lexicographers say it is a proper name for a science founded on a knowledge of bones. So instead of “bone disease” it really means “usage.”	I reasoned that the bone, “Osteon”, was the starting point from which I was to ascertain the cause of pathological conditions, and so I combined the “Osteo” with the “pathy” and had as a result, Osteopathy.
200	…any system of drugs, which is your most deadly enemy. *A doctor will use you for what money he can get out of you.*	…any system of drugs, which is your most deadly enemy.
203	…found at the origin of the gall-producing nerves *in the brain*. Therefore when we are suffering from the effect of *delays in cardiac nerves to forward blood in sufficient quantities to supply cervix,* we have as cause of such pain simply too feeble motion *to start blood to an action of its latent vitality. Thus you have quantity and quality minus motion to the degree of heat by which magnetism can begin the work of vital repairs, or association of the principles of the crude elements of nature, and construct a suitable superstructure in which life can only dwell.*	…found at the origin of the gall-producing nerves. Therefore when we are suffering from the effect of any delay in the nerves to send forward nourishment in sufficient quantities, we have as cause of such pain simply *a* too feeble motion *with which to start blood into action*.
206	The *powers* of lymph are not known. A quantity of blood may be thrown from a ruptured vein or artery and form a large tumefaction *of the parts*, causing a temporary suspension of the vital *there-unto belonging*.	The *functions* of lymph are not known. A quantity of blood may be thrown from a ruptured vein or artery and form a large tumefaction, causing a temporary suspension of the vital forces.
306	God has forgotten nothing, and we find a supply of uric acid *for destroying stone in bladder or gall stones*.	God has forgotten nothing, and we find a supply of uric acid *which will destroy stone in the urinary bladder. His law is equally trustworthy in the destruction of gall stones*.
326	What can you give us in place of drugs? we cannot add or give anything from the material world…	What can you give us in place of drugs? *we can give you adjustment of structure but* we cannot add or give anything from the material world…
326	… substances that have been made so by wear and motion.	… substances that have been made so by wear and motion. *A perfectly adjusted body which will produce pure blood and plenty of it, deliver it on time and in quantity sufficient to supply all demands in the economy of life. This is what the osteopath can give you in the place of drugs if he knows his business.*

**Table 4:** Substantial changes.

Page (1908)	1897	1908
126	*Dr. William Smith, of* Edinburgh, Scotland, came to my house to talk with me and learn something of the *law of cures*,…	*a doctor* from Edinburgh, Scotland, came to my house to talk with me and learn something of the *law*,…
132	…from a competent instructor*, as I believed Dr. William Smith to be at that time.* *Since then he has satisfied me that he is the best living anatomist on earth, his head and scalpel prove that he is as good as the best of any medical college of Europe or America. Since leaving Edinburgh, he has studies and dissected to the extent of the demands of Osteopathy for four years, which makes at least two years further in its qualification for the purpose of remedies. Thus I feel safe in saying that Dr. Smith is to-day the wisest living anatomist on the globe, and will await the successful refutation of the assertion.*	…from a competent instructor.
313	The central figure of the group, *Mother* Sun, illumines space with *her* effulgent rays, and lights the pathway of numerous children and grandchildren too. *She* is a matchless *mother*, and guides *her* children well; each one of them is polished to the highest point of *perfection known to skill*. … in the grand plan which *the mother has* on constant exhibition.	The central figure of the group, *Father* Sun, illumines space with *his* effulgent rays, and lights the pathway of numerous children and grandchildren too. *He* is a matchless *father*, and guides *his* children well; each one of them is polished to the highest point of *perfection*. … in the grand plan which *is* on constant exhibition.
313	Small Mercury dwells close unto *her mother’s* side, as if *she* feared to wander away lest *she* be lost in fields of space. *She* is arrayed in robes of vivid white, without a spot to mar *her* purity.	Small Mercury dwells close unto *his father’s* side, as if *he* feared to wander away lest *he* be lost in fields of space. *He* is arrayed in robes of vivid white, without a spot to mar *his* purity.
313	…to gladden her *Mother’s* heart and help increase the starry progeny. The eldest child of all, *Mrs. Uranus*, … of the old *grandmother*… *Her* family… I saw *the gay, vivacious Mrs*. Saturn, with *her* many rings. *She* smiled on… Moon, *and* shed the light…	…to gladden her *Father’s* heart and help increase the starry progeny. The eldest child of all, *Uranus*, … of the old *grandparent*… *His* family… I saw Saturn, with *his* many rings. *He* smiled on… Moon, that *shed* the light…
314	…of the *lady Sun*, and followed with unfaltering footsteps the line of march *she had laid* out for them. I saw the face of the dear *mother* shrouded by a veil of impenetrable mourning, as if *her* heart were grieved by some erring action of one of *her* beauteous family…and revealed *her* face… *She sent* this message…	…of the *Sun*, and followed with unfaltering footsteps the line of march *laid* out for them. I saw the face of the dear *parent* shrouded by a veil of impenetrable mourning, as if *the* heart were grieved by some erring action of one of *the* beauteous family… and revealed *a* face…*Sending* this message…

**Table 5:** Changes to the pictures.

Page (1908)	1897	1908
Frontispiece	Portrait of Dr. A.T. Still	Different Portrait of Dr. A.T. Still
132	Picture of William Smith, M.D., D.O. (page 154)	Removed
136	Picture “My name is Scarlet Fever; I live on little blue-eyed, fair-skinned children” between pages 160 and 161	Removed
143	“A.T. Still’s Infirmary and school building” (between pages 168 and 169)	Acronym “A.S.O.” added on the roof
147	Picture, portrait: “Mrs. Anne Morris, the amanuensis who wrote this volume according to my dictation” (table between pages 172 and 173)	Removed
206	Picture: “Bust of A.T. Still” (table between pages 250 and 51)	Removed
256	Color picture: “Professor Peacock” (table between pages 316 and 17)	Removed
366	Picture: “It is because you have lied”	Moved from the end of Chapter XXXI to the second page of Chapter XXXII
366	Picture “Muscular System of Man” (Anatomical table between pages 442 and 43)	Removed

## Data Availability

The data presented in this study are available on request from the corresponding author (accurately indicate status).

## Supplementary Materials

The following supporting information can be downloaded at: https://www.mdpi.com/article/10.3390/healthcare12020130/s1, Table S1: "Noteworthy" changes, extracted chapter by chapter.

## References

[1] OIA (2021). The OIA Global Report: Global Review of Osteopathic Medicine and Osteopathy 2020.

[2] Zegarra-Parodi R., Esteves J.E., Lunghi C., Baroni F., Draper-Rodi J., Cerritelli F. (2021). The legacy and implications of the body-mind-spirit osteopathic tenet: A discussion paper evaluating its clinical relevance in contemporary osteopathic care. Int. J. Osteopath. Med..

[3] Gevitz N. (2014). The “doctor of osteopathy”: Expanding the scope of practice. J. Am. Osteopath. Assoc..

[4] Gevitz N. (2014). The “diplomate in osteopathy”: From “school of bones” to “school of medicine”. J. Am. Osteopath. Assoc..

[5] Zegarra-Parodi R., Baroni F., Lunghi C., Dupuis D. (2022). Historical Osteopathic Principles and Practices in Contemporary Care: An Anthropological Perspective to Foster Evidence-Informed and Culturally Sensitive Patient-Centered Care: A Commentary. Healthcare.

[6] Mehl-Madrona L., Conte L.A., Mainguy B. (2023). Indigenous roots of osteopathy. AlterNative Int. J. Indig. Peoples.

[7] Allen T. (1993). Osteopathic physician’ defines our identity. J. Am. Osteopath. Assoc..

[8] Gevitz N. (2014). From “Doctor of Osteopathy” to “Doctor of Osteopathic Medicine”: A Title Change in the Push for Equality. J. Osteopath. Med..

[9] Gevitz N. (2004). The DOs: Osteopathic Medicine in America.

[10] Booth E.R. (1924). History of Osteopathy, and Twentieth-Century Medical Practice.

[11] The Journal of the American Osteopathic Association 1901–1911. https://archive.org/details/sim_jaoa-the-journal-of-the-american-osteopathic-association_1901-11_1_2.

[12] (1902). The Journal of the American Osteopathic Association. https://archive.org/details/sim_jaoa-the-journal-of-the-american-osteopathic-association_1902-11_2_3.

[13] (1903). The Cleveland Meeting. https://archive.org/details/sim_jaoa-the-journal-of-the-american-osteopathic-association_1903-06_2_10.

[14] Varughese H., Shin P. (2010). The Flexner report: Commemoration and reconsideration. Yale J. Biol. Med..

[15] Karle H. (2010). How do we Define a Medical School? Reflections on the occasion of the centennial of the Flexner Report. Sultan Qaboos Univ. Med. J..

[16] Ludmerer K. (2010). Commentary: Understanding the Flexner report. Acad. Med. J. Assoc. Am. Med. Coll..

[17] Coulter I. (1998). Complementary and alternative medicine: A review of the literature. BMJ.

[18] Barnes P.M., Bloom B., Nahin R.L. (2008). Complementary and alternative medicine use among adults and children: United States, 2007. Natl. Health Stat. Rep..

[19] Stahnisch F.W., Verhoef M. (2012). The flexner report of 1910 and its impact on complementary and alternative medicine and psychiatry in north america in the 20th century. Evid. Based Complement. Altern. Med. Ecam.

[20] Gevitz N. (2009). The transformation of osteopathic medical education. Acad. Med. J. Assoc. Am. Med. Coll..

[21] Paul Lee R. (2005). Interface: Mechanisms of Spirit in Osteopathy.

[22] Still C.E., Library of Congress Cataloging–in–Publication Data (1907). The Life and Times of A. T. STILL and His Family.

[23] Stark J. (2007). Still’s Fascia: A Qualitative Investigation to Enrich the Meaning behind Andrew Taylor Still’s Concepts of Fascia.

[24] Are Our Osteopathic Textbooks to Go Out of Print?. The Journal of the American Osteopathic Association 1908–1911: Volume 8, Page 129..

[25] Osteopathic Textbooks The Journal of the American Osteopathic Association 1908–1911: Volume 8, Page 181. https://archive.org/details/sim_jaoa-the-journal-of-the-american-osteopathic-association_1908-12_8_4/page/181/mode/1up?q=books.

[26] Trowbridge C. (1991). Andrew Taylor Still, 1828–1917.

[27] Lewis J.R.A.T. (2012). Still: From the Dry Bone to the Living Man.

[28] Fortún Agud M., Estébanez de Miguel E., Ruiz de Escudero Zapico A., Cabanillas Barea S., Pérez Guillén S., López de Celis C. (2013). La expansión de la Fisioterapia moderna de Ling por el mundo. Cuest. Fisioter. Rev. Univ. Inf. E Investig. Fisioter..

[29] Sutherland A.S. (1962). With Thinking Fingers: The Story of William Garner Sutherland.

[30] Still A.T. (1908). Autobiography of Andrew T. Still: With a History of the Discovery and Development of the Science of Osteopathy, Together With an Account of the…School of Osteopathy.

[31] Medicine MoO (1985). Photo Record #1985.1003.

[32] Medicine MoO (1976). Object Records #1976.131.

[33] ASO (1904). S. S. Twelfth Annual Announcement and Annual Catalogue o the American School of Osteopathy, Kirksville, Missouri, 1904.1905.

[34] Bean A.S. (1919). The theory of osteopathy in nephritic cases. Osteopath. Truth.

[35] Gevitz N. (2014). A degree of difference: The origins of osteopathy and first use of the “DO” designation. J. Am. Osteopath. Assoc..

[36] The Internet Archive. https://archive.org/search?query=autobiography+A.+T.+Still.

[37] Still A.T. (1897). Autobiography of Andrew T. Still: With a History of the Discovery and Development of the Science of Osteopathy, Together With an Account of the…School of Osteopathy.

[38] Azarian R., Petrusenko N. (2011). Historical Comparison Re-considered. Asian Soc. Sci..

[39] Vann R.T. (2023). Historiography Encyclopedia Britannica: @britannica. https://www.britannica.com/contributor/Richard-T-Vann/3043.

[40] May T. (1993). Social Research: Issues, Methods, and Process.

[41] Tilly C. (1984). Big Structures, Large Processes, Huge Comparisons.

[42] Paulus S. (2013). The core principles of osteopathic philosophy. Int. J. Osteopath. Med..

[43] Hildreth A.G. (1938). The Lengthening Shadow of Dr. Andrew Taylor Still.

[44] Medicine MoO (1899). Official KCOM Family about 1898.

[45] Voegelin C.F., Voegelin E.W. (1944). The Shawnee female deity in historical perspective. Am. Anthropol..

[46] Ethridge R., Shuck-Hall S.M. (2009). Mapping the Mississippian Shatter Zone: The Colonial Indian Slave Trade and Regional Insta.

[47] Lucas D.M. (2001). Our Grandmother of the Shawnee Messages of a Female Deity.

[48] Niethammer C. (2010). Daughters of the Earth.

[49] Stabb R. (1994). “When Shawnees Die They Go To Probate Court”: Cultural Practices of the Kansas Shawnees 1830s–1860s. Algonq. Pap.-Arch..

[50] Haxton J. (2016). Andrew Taylor Still: Father of Osteopathic Medicine.

[51] Liem T., Lunghi C. (2023). Reconceptualizing Principles and Models in Osteopathic Care: A Clinical Application of the Integral Theory. Altern. Ther. Health Med..

[52] Zegarra-Parodi R., Draper-Rodi J., Haxton J., Cerritelli F. (2019). The Native American heritage of the body-mind-spirit paradigm in osteopathic principles and practices. Int. J. Osteopath. Med..

[53] Vogel S. (2017). A road to somewhere—Endless debate about the nature of practice, the profession and how we should help patients. Int. J. Osteopath. Med..

[54] Arcuri L., Consorti G., Tramontano M., Petracca M., Esteves J.E., Lunghi C. (2022). “What you feel under your hands”: Exploring professionals’ perspective of somatic dysfunction in osteopathic clinical practice-a qualitative study. Chiropr. Man. Ther..

[55] Leary M.R., Diebels K.J., Davisson E.K., Jongman-Sereno K.P., Isherwood J.C., Raimi K.T., Deffler S.A., Hoyle R.H. (2017). Cognitive and Interpersonal Features of Intellectual Humility. Personal. Soc. Psychol. Bull..

[56] Deffler S.A., Leary M.R., Hoyle R.H. (2016). Knowing what you know: Intellectual humility and judgments of recognition memory. Personal. Individ. Differ..

[57] Thomson O.P., MacMillan A., Draper-Rodi J., Vaucher P., Ménard M., Vaughan B., Morin C., Alvarez G., Sampath K.K., Cerritelli F. (2021). Opposing vaccine hesitancy during the COVID-19 pandemic—A critical commentary and united statement of an international osteopathic research community. Int. J. Osteopath. Med..

[58] Ciardo A., Sanchez M.G., Cobo Fernandez M. (2023). The importance of constructing an osteopathic profession around modern common academic values and avoiding pseudoscience: The Spanish experience. Adv. Integr. Med..

[59] Healy C.J., Brockway M.D., Wilde B.B. (2021). Osteopathic manipulative treatment (OMT) use among osteopathic physicians in the United States. J. Osteopath. Med..

[60] Ward J. (2019). The osteopathic philosophy of health and disease. J. Osteopath. Med..

[61] Rogers F.J., D’Alonzo G.E., Glover J.C., Korr I.M., Osborn G.G., Patterson M.M., Seffinger M.A., Taylor T.E., Willard F. (2002). Proposed tenets of osteopathic medicine and principles for patient care. J. Am. Osteopath. Assoc..

[62] Coulter D. (2018). New dimensions in osteopathic medicine: Emerging perspectives on holism, healing, and the human spirit. J. Am. Osteopath. Assoc..

[63] Esteves J.E., Zegarra-Parodi R., van Dun P., Cerritelli F., Vaucher P. (2020). Models and theoretical frameworks for osteopathic care—A critical view and call for updates and research. Int. J. Osteopath. Med..

[64] Tyreman S., Cymet T. (2012). Osteopathic education: Editorial call for papers. Int. J. Osteopath. Med..

[65] Ballestra E., Battaglino A., Cotella D., Rossettini G., Sanchez-Romero E.A., Villafane J.H. (2022). ¿Influyen las expectativas de los pacientes en el tratamiento conservador de la lumbalgia crónica? Una revisión narrativa (Do patients’ expectations influence conservative treatment in Chronic Low Back Pain? A Narrative Review). Retos.

[66] Rossettini G., Palese A., Geri T., Fiorio M., Colloca L., Testa M. (2018). Physical therapists’ perspectives on using contextual factors in clinical practice: Findings from an Italian national survey. PLoS ONE.

[67] Audoux C.R., Estrada-Barranco C., Martínez-Pozas O., Gozalo-Pascual R., Montaño-Ocaña J., García-Jiménez D., Vicente de Frutos G., Cabezas-Yagüe E., Sánchez Romero E.A. (2023). What Concept of Manual Therapy Is More Effective to Improve Health Status in Women with Fibromyalgia Syndrome? A Study Protocol with Preliminary Results. Int. J. Environ. Res. Public Health.

[68] Saad M., de Medeiros R., Mosini A.C. (2017). Are We Ready for a True Biopsychosocial-Spiritual Model? The Many Meanings of “Spiritual”. Medicines.

[69] Zegarra-Parodi R., Draper-Rodi J., Cerritelli F. (2019). Refining the biopsychosocial model for musculoskeletal practice by introducing religion and spirituality dimensions into the clinical scenario. Int. J. Osteopath. Med..

