# Supplementary material for: The Revisions of the First Autobiography of AT Still, the Founder of Osteopathy, as a Step towards Integration in the American Healthcare System: A Comparative and Historiographic Review
Source: Healthcare (Basel). 2024 Jan 6;12(2):130. doi: 10.3390/healthcare12020130 (PMC10815194; doi:10.3390/healthcare12020130)
Supplement: Supplementary file 1 [file healthcare-12-00130-s001.zip › healthcare-2769432-supplementary.pdf]

Supplementary Table S1 "Noteworthy" changes, extracted chapter by chapter

1.1. Single chapters

1.1.1. Chapter I

Page (1908)	1897	1908
17	I suppose I *bawled*, and filled the bill of nature in the baby life. My mother was as others who had five or six *angels* to yell all night for her comfort.	I suppose I *cried*, and filled the bill of nature in the baby life. My mother was as others who had five or six *children* to yell all night for her comfort.
33	And to-day I am, as I have been for fifty years, fully established in the belief that the artery is the *father of the rivers of life, health, and ease, and its muddy or impure water is first in all disease.*	And to-day I am, as I have been for fifty years, fully established in the belief that the artery is the *river of life, health, and ease, and if muddy or impure disease follows.*

1.1.2. Chapter II

Page (1908)	1897	1908
35	*...full three feet high* when standing	*...near four feet high* when standing
47	He raised his head two feet *in the air, and fixed those basilisk orbs* on me.	He raised his head two feet *above the ground, and fixed his eyes* on me.

1.1.3. Chapter III

Page (1908)	1897	1908
52	He was very much disappointed and disgusted to learn that a professed minister would play a confidence game and rob him of the money *given him by the Tennessee Conference* to support his family while in his missionary work in the wilds of North Missouri	He was very much disappointed and disgusted to learn that a professed minister would play a confidence game and rob him of the money *he had brought with him* to support his family while in his missionary work in the wilds of North Missouri.

1.1.4. Chapter IV

Page (1908)	1897	1908

58	All are leaders in this division of *the greatest war* ever known on earth the war for truth under the banner of Osteopathy	All are leaders in this division of *one of the greatest wars* ever known on earth the war for truth under the banner of Osteopathy
58	This feeling of duty to free all and let each person have an equal chance to so live this life as a part of a vast eternity, preparatory to *joys immortal, which were bought and paid for by the life and blood of the Son of God*, continued to grow	This feeling of duty to free all and let each person have an equal chance to so live this life as a part of a vast eternity, preparatory to *another life*, continued to grow
62	The log was a cottonwood about twenty feet long, *twenty* inches in diameter	The log was a cottonwood about twenty feet long, *thirty* inches in diameter.
65	... he saved my wife's life *in* cholera	.. he saved my wife's life *from an attack of* cholera.
72	I went home to follow the practice of medicine and saw lumber, which I did *1856 to 1860*	I went home to follow the practice of medicine and saw lumber, which I did *until 1860*
72	Then the struggle began, and lasted until he dipped his pen and wrote the golden words: 'Forever free, without regard to race or color'	Then the struggle began, and lasted until he dipped his pen and wrote the golden words: 'Forever free, without regard to race or color,' *I will add – or sex.*

1.1.5. Chapter V

Page (1908)	1897	1908
73	We were men who meant business and had started out to do some very severe and successful fighting. *We declared that our canteens were to catch rebel blood instead of carry water*. From Leavenworth we were ordered to Kansas City to complete our outfit, and were placed in the brigade of James H. Lane, then commissioned to organize the Western army	We were men who meant business and had started out to do some very severe and successful fighting. From Leavenworth we were ordered to Kansas City to complete our outfit, and were placed in the brigade of James H. Lane, then commissioned to organize the Western army
73	Though we did not come in sight of the Confederates during the march, we *had the satisfaction of tearing down* many flags...	Though we did not come in sight of the Confederates during the march, we *took down* many flags...
74	I counted sixty-two fresh graves in one grave yard, near Harrisonville*, which were said to be the graves of rebels killed on that occasion.*	I counted sixty-two fresh graves in one grave yard, near Harrisonville.
75	A few months later there came another order to consolidate with some other battalions, by which I was *transferred to major* of the Twenty-first Kansas militia.	A few months later there came another order to consolidate with some other battalions, by which I was *transferred, and commissioned major* of the Twenty-first Kansas militia.
76	The Twenty-first Kansas nobly held its ground while we were *bathed in* fire, smoke, and blood.	The Twenty-first Kansas nobly held its ground while we were *surrounded by* fire, smoke, and blood.

1.1.6. Chapter VI

Page (1908)	1897	1908

84	Day and night, *like any other grave-robber*, I roamed about the country, *and* often at moonlight and often in the day-time with shovel disinterred the dead Indian and utilized his body for the good of science.	Day and night I roamed about the country, often at moonlight and often in the day-time with shovel disinterred the dead Indian and utilized his body for the good of science.
85	I shall not allow my equanimity of mind to be disturbed by the thoughts that I once *was a grave-robber*.	I shall not allow my equanimity of mind to be disturbed by the thoughts that I once *sought knowledge from Indian bones*
85	"...and after the Civil War, until like a burst of sunshine the whole truth dawned on my mind, that I was gradually approaching a science by study, research, and observation that *the world is receiving*.	"...and after the Civil War, until *on June 22nd, 1874,* like a burst of sunshine the whole truth dawned on my mind, that I was gradually approaching a science by study, research, and observation that *would be a great benefit to the world.*
88	I have never failed to find all remedies in plain view on the front shelves *of the store of the Infinite.*	I have never failed to find all remedies in plain view on the front shelves *and in the store house of the Infinite—the human body*
89	Man should study and use the drugs *of his drug-store only.*	Man should study and use the drugs *compounded in his own body.*

1.1.7. Chapter VII

Page (1908)	1897	1908
92	My family was small, my wife was *sharp*, and I had to churn.	My family was small, my wife was *busy*, and I had to churn.
93	I could easily get a motion of the cups equal to five hundred or *thousand* revolutions per minute	I could easily get a motion of the cups equal to five hundred or *more* revolutions per minute
93	This year I began an extended study of the drive-wheels, pinions, cups, arms, and shafts of life, with their forces...	This year I began an extended study of the drive-wheels, pinions, cups, arms, and shafts of *human* life, with their forces...
93-94	...voluntary and involuntary nerves in performing their duties, the source of *supplies*, and the work *being done* in health, in the obstructing parts, in the places, *and principles*, through which they passed to perform their part *of the functions of life; all awoke a new interest in* me.	...voluntary and involuntary nerves in performing their duties, the source of *their supply*, and the work *done* in health, in the obstructing parts, in the places, through which they passed to perform their part *in the economy of life; all this study awoke a new interest within* me.
94	... a temporary or permanent suspension of the blood either in arteries or veins, *which effect caused* disease.	...a temporary or permanent suspension of the blood either in arteries or veins, *and cause* disease.
94	Day by day the evidences grow stronger and stronger that this *theory* is correct.	Day by day the evidences grow stronger and stronger that this *philosophy* is correct.
94	For *twenty-three* years it has withstood the storms, cyclones, and blizzards of opposition.	For *twenty-five* years it has withstood the storms, cyclones, and blizzards of opposition.

1.1.8. Chapter VIII

Page (1908)	1897	1908
96	I determined to try my luck *with what I then thought to be a new discovery.*	I determined to try my luck *in the introduction of what I had proven to be a new discovery and a remedy for human ills.*
96	and was appointed by the commissioners of the general conference as agent with my brother	and was appointed by the commissioners of the general conference as agent with my brother Thomas, J. B. Abbott, *Dan'l Fry*, James Blood,

	Thomas, J. B. Abbott, James Blood, and others, to select and locate a spot for the university building.	and others, to select and locate a spot for the university building.
97	and doctoring the sick through small-pox, cholera, and *all the fevers*,	and doctoring the sick through small-pox, cholera, and *other diseases*,
98	I believed Jim's brain would ripen in time, so *just* let him pray,	I believed Jim's brain would ripen in time, so *I* let him pray,
99	...and regrets that his mind was so far below high-water mark, *when it was held up to the* mental feast,	...and *he* regrets that his mind was so far below high-water mark, *that he could not see its perfection as a healing art, when it was held up to his view that he might have a* mental feast,
99	My first case was pneumonia of both lungs.	My first case was pneumonia of both lungs *in an advanced stage*.
99	Hiram Kepner came with *a pair of purulent sore eyes, having ulcerated iris of both.* [..] I simply *used the* blood of the nutrient arteries *only*.	Hiram Kepner came with *purulent sore eyes, of the worst kind.* [..] I simply *brought the arterial* blood of the nutrient arteries *to the eyes to do the work of repair*.
100	...treated her strictly by the *teachings* of Osteopathy, and she was well in thirty-six hours. I have since treated a great number of cases of erysipelas by this *law* and cured all.	...treated her strictly by the *rule* of Osteopathy, and she was well in thirty-six hours. I have since treated a great number of cases of erysipelas by this *method* and cured all *of them*.
100	.. four doctors had used *four ounces of* chloroform on him, ...	...four doctors had used chloroform on him, ...
100	I have never failed on a case of asthma to date, and after *eighteen* years' practice...	I have never failed on a case of asthma to date, and after *many* years' practice...
101	A few months later *I found a man in great distress with asthma. I got off my horse and "hoodledooed"* him.	A few months later *as I was driving across the country on business, I found a man in great distress, suffering with an attack of asthma. The day was cold but the man sat out of doors astride a chair with his face to the back of it; he was gasping for breath and suffering so much that his family, helpless to relieve him, stood around him crying. I quickly dismounted and "hoodledooed" him, or in other words, I treated him, giving him relief at once, and he has had no return of the asthma during the six years which have passed since the treatment was given* him.
101	She had heard that it was faith cure, Christian science, spiritualism, and a great many *kinds of names.*	She had heard that it was faith cure, Christian science, spiritualism, and a great many *other kind of cures.*
103	It *began and terminated with* a painless birth of an eight-pound boy baby, in something less than one hour from the first sign of labor. This was possibly the twentieth case delivered by this method, which I consider worth all the midwifery written to date.	It *was* a painless birth of an eight-pound boy baby, in something less than one hour from the first sign of labor. This was possibly the twentieth case delivered by this method, which I consider worth all the *knowledge of* midwifery written to date *by the old schools.*

1.1.9. Chapter IX

Page (1908)	1897	1908
105	I began to reason, for I then knew very little about flux, more than that it killed young and old, and was worse *in Kentucky in warm weather than in some other States.*	I began to reason, for I then knew very little about flux, more than *the fact* that it killed young and old, and was worse *in warm weather*.
105	...could not get their eyes off the effects *rather than* cause.	...could not get their eyes off the effects *to turn them to* the cause.

107	On examination I found the atlas or *first joint* of her neck...	On examination I found the atlas or *the first bone* of her neck...
108	They weep and mourn because they did not know a *truth from a lie*, ...	They weep and mourn because they did not know a *true philosophy*, ...
110	Man naturally fears that which he does not understand.	Man naturally *dreads to travel a road he has never been over and* fears that which he does not understand.
110-111	To-day Kirksville has a population of *eight* thousand...	To-day Kirksville has a population of *six* thousand...
111	His mother (Mrs. Truit) *carried* him to me...	His mother (Mrs. Truit) *brought* him to me...
111	... because some *gimlet-eyed blatherskite* had told him that Still was a crazy crank,...	... because some old *narrow minded person* had told him that Still was a crazy crank,...
112	So *in 1877* I gave up traveling...	So I gave up traveling...
112	I will conclude this chapter with an *amusing scientific incident...*	I will conclude this chapter with an *incident...*
112	Let us go *in the saloon and have something to take!" He was in his shirt-sleeves, with an abdomen as large as a full moon hung to him, from which I thought he had had too much "to take." In a joking way I exposed about a half-acre of his abdomen on the public street before hundreds of people, and said:* "My dear friend, I have power on earth and in heaven. I am acquainted with the living men and angels, male and female, says...	Let us go *into the saloon and have something to drink." I was not a whisky drinker, and I knew by this man's appearance that he had already had too much. He was in his shirt-sleeves, and his large abdomen extended out prominently. With a hope that I might benefit him, yet in a joking way, I exposed a portion of his abdomen, right there, on the public street and before a large number of people, and putting on a serious tone said to him:* "My dear friend, I have power on earth and in heaven. I am acquainted with the living men and angels, male and female, *and your mother, whose spirit right now overshadows you*, says...
113	I *put my hand upon his abdomen, punched, snatched, and scratched, and told the old gentleman that, "From this day on whisky will make you sick. It will make you vomit whenever you smell of it. If you think I lie, go stick your nose in that saloon, and come back to me." In a few minutes he returned, and said that he got the smell of the beer and whisky, and he began to turn sick at the stomach. He didn't want to stay any longer for fear he would throw up. I watched his conduct for a period of seven years, at which time he died, having never tasted whisky from the time I told him I knew all about devils, life, and death, and he always thanked me for rescuing him from drunkenness. He made an effort to pass the saloons three times a day, which he had entered and spent sixty cents daily for over twenty years, according to his own statement. His wife being a Christian woman, on learning that I was the man who saved her husband from drunkenness, whenever she met me greeted me with, "God bless Brother Still!"*<br>*I had no object in view when I pow-wowed the old gentleman, punched and twisted his abdomen, and told him of the awful ending of the sot, except a little street fun. What I considered nonsensical and foolish had the effect to make a sober man of him, and saved sixty cents each day out of his daily labor for his good wife to apply in the necessaries and comforts of life. I never told the old man nor his wife that all that pow-wow was simply a little*	I *then began with my hands upon his abdomen, to push and pull and rub and twist, then I worked his spine and ribs. After that I put my elbow in his back and pulled him backwards over it with force. My object was to get the old man's mind off the thought of the drink until I could impress him with my words which were as follows: "From this day forth whiskey will make you sick. You will want to vomit every time you smell it." After a while I told him to go to the saloon smell the whiskey, then come back to me and if he was not sick of the smell of it I would pay for his drink he went to the saloon and came back shortly telling me that he felt sick at his stomach and had no desire to take a drink. This was my first effort with the drink habit, and I confess my surprise to learn, as the years passed by, that the man had entirely lost his desire for whisky. His wife, a Christian woman, on learning that I was the man who saved her husband from drunkenness, would greet me whenever she met me with, "God Bless Brother Still." Three times daily this old man would pass by the saloons in which he had been in the habit of spending sixty cents a day for over twenty years. He always thanked me for rescuing him from drunkenness. About seven years later he died a sober man.*<br>*I thought but little of the philosophy of it, or why a person should have such a desire for alcoholic drinks, but after his death I thought about it and reasoned that a failure of the pancreas, spleen, or*

	nonsense, because I saw they both believed I was a heavenly messenger, and through me the angels had saved her husband. Some other ladies brought a doctor to me. One held to each arm, trying to beguile him...	*liver to perform their natural functions and generate compounds in sufficient quantities to neutralize the fluids which by nature should be kept harmonious and satisfy that demand without the alcohol, was the cause.*<br>*This man's case, the treatment and its result, has led me to experiment with others with good results. I believe the cause of drunkenness is a failure of these organs to carry on their normal functions.*<br>*I began to soliloquize and question along this line. Is it a disgrace for a man to drink alcohol, brandy or whisky when he has a great thirst for such drinks? Would it not be cruel to turn that man coldly away as though he were a criminal and you would be disgraced if seen in his company? Is he diseased? Does his drinking to beastly drunkenness cry in thunder tones to any philosopher who can reason from effect to cause, to halt and stay with this man and never leave him until he has found and removed the cause which produced this thirst for the liquor which has been (in his opinion)—the drunkard's only friend, to care for and comfort the lonely hours of his affliction, while it has at the same time ruined his home and all the joys of his life and those of his loved ones. Was that whisky his friend? I say yes, a thousand times. But the Doctor did not hear the cry nor see the finger of nature in this man's craving for whisky, pointing to the cause of this insatiable thirst for liquor as being a failure of the organs of the body to deliver their secretions in quantity and quality sufficient to hold the chalk, lime, and other earthy substances in solution and by so doing prevent the formation of gall or kidney stones. Is it not within reason to conclude that the alcohol drinker is a sick man? One whose acts tell any reasoner that he has had by accident, strain or otherwise a suspension of nerve and blood supply to the pancreas, spleen or liver, and that the desire for alcohol will disappear after an adjustment of structures such as will admit of a normal nerve and blood supply to these organs?*<br>*In speaking of whisky being the drunkard's greatest friend we will explain by saying, that while it gave him temporary relief from the oppressive action of the lime and chalk which was retained in his system in an abnormal condition, it should be the Doctor's greatest friend, as well as an aid in his diagnosis, if he has any power to reason, from effect to cause, and when he finds a temporary paralysis of the nerves of the spleen, pancreas, or liver to such a degree as to suspend the generative powers of those organs or in any way interfere with the production of acids in sufficient quantities to neutralize the chalk and lime de- posits in the system, he knows full well (providing his Osteopathic principles are well grounded) how to take off the pressure or remove the obstruction or*

		make the structural adjustment necessary to restore the organs to their normal functioning. Shortly after I had treated the old gentleman some ladies brought a doctor to me to be treated for the drink habit. One held to each arm, trying to beguile him...
116	Suppose I had relieved this doctor of this thirst for whisky, *fixed* a few more, and had got something like a popular craze among the doctors to be treated for the whisky habit, how many hundred thousands would I have had to *punch and spank and scratch* each year?	Suppose I had relieved this doctor of this thirst for whisky, *treated* a few more, and had got something like a popular craze among the doctors to be treated for the whisky habit, how many hundred thousands would I have had to *punch, pull and twist* each year?

1.1.10. Chapter X

Page (1908)	1897	1908
119	but the cries of our children call a halt to *such a thought, and the deadly drug and knife of the suicide are cast into the fire. I have long thought I might at some time be called to stop my useless life of misery and hours of lamentations.*<br>*With trembling gait my wife* came to my side...	but the cries of our children call a halt to *the thought of the deadly drug and knife of suicide. In a vision of the night of despair, I saw my wife who* came to my side...
119	I *listened* to his little story, and when he said he hunted and hunted all alone till he found work, like a flash of lightning I *saw* hope and joy perched on a stone, *with all that man could hope or wish for.*<br>I saw the brain of the man of success on a dish and a great golden *plate or banner* floating to the breeze. *At the top of the plate I saw a picture of* a man's brain...	I *seemed to listen* to his little story, and when he said he *had* hunted and hunted all alone till he found work, like a flash of lightning I *seemed to see a picture of* hope and joy perched on a stone. I *saw in my vision the brain of the* man of success on a dish and a great golden banner floating to the breeze. On the plate I saw a man's brain...
119	This is of no use to *others*, it is no better than others only in one way, *he* had the courage to use *it* and let all others alone.	This is of no use to *any other man*, it is no better than others only in one way, *this man* had the courage to use *his own brain* and let all others alone.
120	...one's brain is his only *reliance*. [...] It is the judge that *God* sends...	...one's brain is his only *reliable friend*. [...] It is the judge that *Nature's architect* sends...
120	... for those who depend on *Him*?	... for those who depend on *them*?
120	his own brain, which *is* his compass and quadrant for *his* vessel,...	his own brain, which *should be* his compass and quadrant for *the* vessel,...
121	But his wife, the faithful friend, says: [...] she rouses herself with a *superhuman* energy [...] her brain is her only store house, and from it must the milk *and her supplies* be drawn.	But his wife, the faithful friend, says: *(as is often the case)*, [...] she rouses herself with a *mother's* energy [...] her brain is her only store house, and from it must the milk *of energy and intelligence* be drawn.
124	...you have served in this war, in *all ranks* from private to general, and I wish your name placed on the retired list.	...you have served in this war, in *every rank* from private to *major* general, and I wish your name placed on the retired list.

1.1.11. Chapter XI

Page (1908)	1897	1908
126	It was not quite as hard as *bull's, for in about twelve months I got* a few ideas in...	It was not quite as hard *as a diamond nor near as brilliant. In about twelve months I succeeded in getting* a few ideas into...
126	*Dr. William Smith, of* Edinburgh, Scotland, came to my house to talk with me and learn something of the *law of cures,...*	*a doctor* from Edinburgh, Scotland, came to my house to talk with me and learn something of the *law,...*
127	I am a graduate of medicine *of seven years* from Edinburgh,	I am a graduate of medicine from Edinburgh,
127	...childbirth without pain, *taking off* goitres, in pneumonia,	...childbirth without pain, *reduction of* goitres, in pneumonia,
127	I was a doctor of medicine of *seven* years' drill in Edinburgh.	I was a doctor of medicine of *five* years' drill in Edinburgh.
127	I had met *Dr. Smith...*	I had met *this doctor...*
128	... the motor and sensory.	... the motor and sensory *or the positive and negative.*
130-131	"You have discovered that which all philosophers have sought for two thousand years," adding: "I am no fool, and as a doctor of medicine I have read all history and know such was never known before.	"You have discovered that *for* which all philosophers have sought for two thousand years *and have failed to find,*" adding: "I am no fool, and as a doctor of medicine I have read all history and know *that* such *philosophy* was never known before.
131	The class advanced *as far as all* of the bones and muscles of the arm and leg. A few of *that* class did not return to finish...	The class advanced *only as far as the study* of the bones and muscles of the arm and leg. A few of *the* class did not return *after that four-months term* to finish...
131	I took the class *after Dr Smith* had stopped at the study of the bones of the body and muscles of the arm and leg *only*. I could get a few ideas in their heads when I talked about a leg or arm, but *could advance them no further.*	I took the class *which* had stopped at the study of the bones of the body and muscles of the arm and leg. I could get a few ideas in their heads when I talked about a leg or *an* arm, but *everything was Greek to them beyond tha*t.
132	This *imperfect* knowledge created a desire to go into the world as cure-alls and know-alls, *who want* to say and write all and much more than is in Osteopathy.	This *limited* knowledge created a desire *on their part* to go *out* into the world as "cure-alls and know-alls," *wanting* to say and write all and much more than is in Osteopathy
132	...from a competent instructor*, as I believed Dr. William Smith to be at that time. Since then he has satisfied me that he is the best living anatomist on earth, his head and scalpel prove that he is as good as the best of any medical college of Europe or America. Since leaving Edinburgh, he has studies and dissected to the extent of the demands of Osteopathy for four years, which makes at least two years further in its qualification for the purpose of remedies. Thus I feel safe in saying that Dr. Smith is to-day the wisest living anatomist on the globe, and will await the successful refutation of the assertion.*	...from a competent instructor.
132	Picture of Dr. Smith removed	
133	They know it falls to their lot to bear all the suffering and lacerations; therefore it is reasonable to suppose, for the sake of their sex, they will continue *the study of the laws* of parturition *to* a comprehensive and practical knowledge of all the principles belonging to this branch of Osteopathy.	They know it falls to their lot to bear all the suffering and lacerations *received through the ignorance of the doctor*; therefore it is reasonable to suppose, for the sake of their sex, they will continue *to study the. law* of parturition *and gain* a comprehensive and practical knowledge of all the principles belonging to this branch of Osteopathy, *which teaches that lacerations to the mother and*

		injury to the child by forceps are not necessary except in extreme cases of bone deformities.
139	In this area of reason, you have a circle that contains only *supposable* facts.	In this area of reason, you have a circle that contains only facts.
140	If he did he would be like *a man* who had his head in many kinds of *business at the same time*, and fails because he cannot climb but one tree at a time.<br>He is not the man to put *at the head of your business*. He has too many ideas, and may do for you as he has for himself, prove *he has failed, fall, and pull you down too.*	If he did he would be like *an osteopath* who had his head in many kinds of *cures*, and fails because he cannot climb but one tree at a time.<br>He is not the man to put *in your sick room*. He has too many ideas, and may do for you as he has *done* for himself, prove *that he is a failure in all places*.
144	*Blanche* Still	*Branche* Still
146	George *R.* Rupe	George *W.* Rupe

1.1.12. Chapter XII

Page (1908)	1897	1908
149	that grand being containing Mind, Matter, and Motion, and given in form "and endowed with the attributes of *God", which* he wants to live on and on forever.	that grand being containing Mind, Matter, and Motion, and given in form *of human being or man* "and endowed with the attributes of *Divine Intelligence"*, he wants to live on and on forever.
149	Until the birth of Osteopathy, [...] death has *never* lost a single victory...	Until the birth of Osteopathy [...] death has *seldom* lost a single victory...
150	I have explored by reading and inquiry *all* that has been written...	I have explored by reading and inquiry *much* that has been written...
151	"And shall I travel the lonely road of another *eternity* and not be noticed by man?"	"And shall I travel the lonely road of another *century* and not be noticed by man?"
156	In short, you are advocating a system that is unnatural and destructive to life, and the world *would be* better off without you.	In short, you are advocating a system that is unnatural and destructive to life, and the world *is* better off without you.
159	...that arterial action has been increased by heat to such velocity that veins cannot return *blood. Contract veins, and stop* the equality of exchange between veins and arteries.	...that arterial action has been increased by *sun*-heat to such velocity that veins cannot return *blood normally, but they become contracted, stopping* the equality of exchange between veins and arteries. *Then a chill follows for a short time, then fever.*
161	...the great table that set in the center of the University of Deity, covered all over with the finest *fruits, each one equal to the finest gem.*	...the great table that set in the center of the University of Deity, covered all over with the finest *fruit.*
162	"Forever free, without regard to race or color."	"Forever free, without regard to race or color." *I amend and add sex.*
163	We are not enrolled under the banner of a *theologian.*	We are not enrolled under the banner of a *theorist.*
164	... coming down as from the very *bosom* of God Himself. All this *fertility* we believe is intended for the human race and for the benefit of man. With the power of production found in *this soil,...*	...coming down as from the very *mind* of God Himself. All this *richness* we believe is intended for the human race and for the benefit of man. With the power of production found in *the soil of this new country,...*
164	... without a *perfect* knowledge of anatomy...	... without a *good* knowledge of anatomy...

1.1.13. Chapter XIII

Page (1908)	1897	1908
169	...for a thousand *ages*.	...for a thousand *years*.
170	...the way to his great and red-hot *black* reward.	...the way to his great and red-hot reward.
170	...opium, calomel, quinine, jallop, ...	...opium, calomel, *whisky*, quinine, jallop, ...
173	...and ask you to keep it *out of my special* opinion in this case,...	...and ask you to keep it *private and out of my written* opinion in this case,...
174	...and it is *love* to all mankind,...	...and it is *Love* to all mankind,...
175	I was met at the door by the sexton,...	I was met at the door by the janitor,...

1.1.14. Chapter XIV

Page (1908)	1897	1908
178	...I have been visited by *the visions of* the night...	...I have been visited by *visions in* the night...
181	...*stating* eighty births in Chicago, in which the forceps were used *in all, and lacerations occurred in forty-three from one to more inches, all of which* will have to go under chloroform for surgical operation.	...*reporting* eighty births in Chicago, in which the forceps were used *and laceration of one or more inches occurred in forty-three cases, all of these patients* will have to go under chloroform for surgical operation.
182	He who wished to successfully solve the problem of disease or *deformities* of any *kinds in all cases* without exception would find one or more *obstruction* in some artery, *or some of its branches.*	He who wished to successfully solve the problem of disease or *deformity* of any *kind in every case* without exception would find one or more *obstructions* in some artery, *or vein.*
182	The rule of the artery *must be* absolute, universal, and unobstructed, or disease *will be the* result.	The rule of the artery *is* absolute, universal, and *it must be* unobstructed, or disease *will* result.
182	...further proclaimed that the *brain* of man was God's drug-store...	...further proclaimed that the *body* of man was God's drug-store...
183	*No discovery is newer than twenty-four years of this science, as shown by any one to date.*	*I made this discovery more than twenty-four years ago.*
184	Greek lexicographers say it is a proper name for a science founded on a knowledge of bones. So instead of “bone disease” it really means “usage.”	I reasoned that the bone, "Osteon," was the starting point from which I was to ascertain the cause of pathological conditions, and so I combined the "Osteo" with the "pathy" and had as a result, Osteopathy.
185	*God* would not be forgetful [...] and there is much evidence that *mind* is imparted to the corpuscles of the blood...	*Nature* would not be forgetful [...] and there is much evidence that *knowledge* is imparted to the corpuscles of the blood ...
186	You dare not assert that *the Deity*...	You dare not assert that *God*...

1.1.15. Chapter XV

Page (1908)	1897	1908
192	...under the rigors of a foreman *that receives* nothing but perfection in constructing a temple is evident — to *receive* in workmanlike order a dwelling-place for the *spirit-man* in such perfect running order that the indweller may be able to operate the machinery easily at all times, *is evident.*	...under the rigors of a foreman *who conceives* nothing but perfection in constructing a temple is evident — to *construct* in workmanlike order a dwelling-place for the *spirit of man* in such perfect running order that the indweller may be able to operate the machinery easily at all times.
193	...when driven by the power of *life at the command of God, who gives power to all elements of force that exist beneath the great throne of mind...*	...when driven by the power of *life, which controls all the elements of force that exis*t...
193-194	We find two large and complete systems of vessels called the *channels of blood, through which to and from a great reservoir containing the fluids* are [...]. We trace *from this great tank* to another fountain of supplies [...]and delivered to the heart, to be sent [...]. Thus we *hear the appellation* the "blood of life"...	We find two large and complete systems of vessels called the blood channels, *through which to and from the heart flows the fluid, they* are [...]. We trace *the blood from the heart* to another fountain of supplies [...]and delivered to the heart *and lungs*, to be sent [...]. Thus we *have* the "blood of life"...
194	... in every *minutia* to the common whole,...	... in every *particular* to the common whole,...
196	I will use my pen and feed the coming minds the best I can. *So I must say farewell as a physical engineer.*	I will use my pen and feed the coming minds the best I can.
197	... by the mind of an unerring *God.*	... by the mind of an unerring *Architect.*
199	...a cause in the spinal cord or *other nerves,...*	...a cause in the spinal cord or *its branches,...*
199	...the power of nature to cure is *false, or else they do not understand their business.*	...the power of nature to cure is *false.*
200	...any system of drugs, which is your most deadly enemy. *A doctor will use you for what money he can get out of you.*	...any system of drugs, which is your most deadly enemy.

1.1.16. Chapter XVI

Page (1908)	1897	1908
201	An absolute demand for *revolution* is before us...	An absolute demand for *reformation* is before us...
202	He Himself cannot succeed without a close observance of the *laws of success,...*	He Himself cannot succeed without a close observance of the *laws*, ...
202	The kind and quantity must be supplied at the right time and place only. If this fluid be in the brain, open the rivers and they will expel all driftwood and *unkindly* substances, and at once to the duties of their division, which is *life with all its harmony. That division is law* and life itself.	The kind and quantity *of fluid or force* must be supplied at the right time and place only. If this fluid be in the brain, open the rivers and they will expel all driftwood and *waste* substances, *and permit the organs to proceed* at once to the duties of their division, which is law and life itself.
202	Death is *completed work of development of the sum total of effect to a finished work of nature.*	Death is the *end or the sum total of effects.*
203	...found at the origin of the gall-producing nerves *in the brain.* Therefore when we are suffering from the effect of *delays in cardiac nerves to forward*	...found at the origin of the gall-producing nerves. Therefore when we are suffering from the effect of any delay in the nerves to send forward

	blood in sufficient quantities to supply cervix, we have as cause of such pain simply too feeble motion *to start blood to an action of its latent vitality. Thus you have quantity and quality minus motion to the degree of heat by which magnetism can begin the work of vital repairs, or association of the principles of the crude elements of nature, and construct a suitable superstructure in which life can only dwell.*	nourishment in sufficient quantities, we have as cause of such pain simply *a* too feeble motion *with which to start blood into action.*
204	The commander of my store of wisdom has for once called a halt, as I *view* one of the most mysterious and beautiful *sights of my life* – the working of the Grand Architect...	The commander of my store of wisdom has for once called a halt, as I *try to comprehend* one of the most mysterious and beautiful *works* – the working of the Grand Architect...
205	... the building of a thigh-bone, perfect in all *material and mental* parts.	... the building of a thigh-bone, perfect in all *its* parts.
206	The *powers* of lymph are not known. A quantity of blood may be thrown from a ruptured vein or artery and form a large tumefaction *of the parts*, causing a temporary suspension of the vital *there-unto belonging*.	The *functions* of lymph are not known. A quantity of blood may be thrown from a ruptured vein or artery and form a large tumefaction, causing a temporary suspension of the vital forces.
206	...conducted by the skilful plan of *that principle of mind commonly known as God, which has* the power...	...conducted by the skilful plan of *God, with* the power...
207	*Anxious nature* stands fully armed and equipped, ...	*Nature* stands fully armed and equipped, ...
208	... angels and worlds, are atoms *of which you are composed.* [...] Therefore be kind in thought to the atoms of life*, or in death you will be borne to the grave by the beasts of burden who carry nothing to the tombs but the bodies of heedless stupidity, the mourners being the asses who cry and bray over the loss of their dear brother.*	... angels and worlds, are atoms. [...] Therefore be kind in thought to the atoms of life.
209	Let us reason with a *faith* that nature does know...	Let us reason with a *thought* that nature does know...

1.1.17. Chapter XVII

Page (1908)	1897	1908
211	Small seeds and other substances have been found in the vermiform appendix, which is a hollow tube *over an inch* in length.	Small seeds and other substances have *in some few cases* been found in the vermiform appendix, which is a hollow tube *from one to four inches* in length.
212	The bowels are rolled over and around in the search *of the appendix*. Sometimes *some* substances are found in it;...	The bowels are rolled over and around in the search. Sometimes substances are found in it;...
214	...and so wisely furnished by *Deity* as to ferret out disease,...	...and so wisely furnished by *God* as *to be able* to ferret out disease,...
214	...and ignorance so *adjusted his mind to submit to customs* of the great past...	...and ignorance so *accustomed his mind to submit to the teachings* of the great past...
215	With all your wisdom can you improve on His hair-making *machine*?	With all your wisdom can you improve on His hair-making?

216	...his eye comes in contact with new worlds *dead and alive*;	...his eye comes in contact with new worlds;
217	To hold it open is the *token* of death or of usefulness.	To hold it open is the *symbol* of death or of usefulness.
218	Each of them represents a chemical or soul quality of a *distinct* division of the great laboratory of nature.	Each of them represents a chemical or soul quality of a *district* division of the great laboratory of nature.
220	When it moves, you *know*. When it stops, you see the end.	When it moves, you *live*. When it stops, you see the end.

1.1.18. Chapter XVIII

Page (1908)	1897	1908
222	My father's salary the first year was the munificent sum of *$6*.	My father's salary the first year was the munificent sum of *$60.00*.
222	...fears being *kicked out of* the lunatic asylum	...fears being *assigned to* the lunatic asylum
222	...the new *discoveries* I hope to make in this science by that time.	...the new *developments* I hope to make in this science by that time.
223	You see one little *lie* always calls for *more* to *cover* it up.	You see one little *excuse* always calls for *others* to *bolster* it up.
223	Activity of the Osteopathic student begins...	*Mental* activity of the Osteopathic student begins...
225	...its victims dying, at a *rate of one hundred and fourteen per day*...	...its victims dying, at a *great rate*...
226	*It may be that the whole* masses are not Galileos,...	*The* masses are not Galileos,...
227	I felt very timid, because I didn't know how *little* sense he had, nor how *much*. I had seen a glimpse of what I considered the *very candle of God Himself*, lighted and sustained by the oil of reason. *The speaker said: "Now,* Mr. Harris, if you will arise I will show this people just the size of Osteopathy then." *(Mr. Harris appeared on the platform). If* you examine...	I felt very timid, because I didn't know how *much* sense he had, nor how *little*, *but* I had seen in *him* a glimpse of what I considered the *candle of God*, lighted and sustained by the oil of reason. *I will now introduce this mechanic to you.* Mr. Harris, if you will arise I will show this people just the size of Osteopathy then. *Now, if* you examine...
228	...I see *the deltoid or any other muscle* in position ...	...I see *muscle* in position ...
228	None of His *children* disobey,...	None of His *worlds* disobey,...
229	... endows him with sense *plentiful* enough to suffice...	... endows him with sense enough to suffice...

1.1.19. Chapter XIX

Page (1908)	1897	1908
232	Osteopathy – a drugless science – finds the utero-genital nerves *made tight by the fastening of certain segments*.	Osteopathy – a drugless science – finds the utero-genital nerves *deranged by irritation*.
234	...go home and take *half a glass of castor oil* and purge yourself of such notions.	...go home and take *a dose of reason* and purge yourself of such notions.

	If you consider me a mesmerist, a big dose of *pills* may carry that thought away.	If you consider me a mesmerist, a big dose of *anatomy* may carry that thought away.
235	Shut it off in one place and congestion *may result*,...	Shut it off in one place and congestion *results*,...
236	... unless he found on removing his hide marks of previous bodily injury, *not of hi choice.*	... unless he found on removing his hide marks of previous bodily injury.
236-238		Poem OSTEOPATHY by Helen de Lendrecie added.

1.1.20. Chapter XX

Page (1908)	1897	1908
239	Have I a mind capable of comprehending or solving by my *force of philosophy* the great question,...	Have I a mind capable of comprehending or solving by my *philosophy* the great question,...
240	...and all the aspirations of *God Himself in kind.* All these qualities you find in man. The same qualities you find in a more refined *condition* in woman,...	...and all the aspirations of *God.* All these qualities you find in man. The same qualities you find in a more refined *form* in woman,...
244	It has absolute control over the nervous system of the lungs, and if *there is no pocket or cavity made in them, I believe* the law is absolute...	It has absolute control over the nervous system of the lungs, and if *no pocket or cavity has been formed in them, the* law is absolute...
244	*Mr. Dunglison*, will you please explain to the people what headache is? [...] Here is your definition of headache by *Dunglison.*	*Dr. Medic*, will you please explain to the people what headache is? [...] Here is your definition of headache by Mr. *Medic.*
245	Nature's God, in constructing that house, [...] Are the *wires* all in place [...]<br>...when they are all in their normal position *a normal God has declared it* is in proper condition for health.	Nature's God, in constructing that house *not made with hands*, [...] Are the *"pipes"* all in place [...]<br>...when they are *all* in their normal position *that every part* is in proper condition for health."
248	...of God *Himself.*	...of God.
248	...principles that the *Divine Mind* intended should be in man.	...principles that the *Architect* intended should be in man.

1.1.21. Chapter XXI

Page (1908)	1897	1908
249	...a science which any man... can *learn.*	...a science which any man... can *comprehend.*
251	...to the study of these *human* engines...	...to the study of these engines...
254	... to follow the *sexton* [2 recurrencies]	... to follow the *janitor* [2 recurrencies]
256	...to forming a being called the feather*, coming out of the back of any fowl.*	...to forming a being called the feather.

1.1.22. Chapter XXII

Page (1908)	1897	1908
258	...to meditate upon that event, *upon that day,* wherein I saw...	...to meditate upon that event, wherein I saw...
261	...go out into the world and raise *his flag of "Osteopathy".*	...go out into the world and raise *their flags and call themselves "Osteopaths".*
264	...the father and mother were also dead *of flux.*	...the father and mother were also dead.
265	One of my poor, *feeble-minded* sons, who has...	One of my poor, *"feeble-minded"* sons, who has...
266	He also told me, *previous to that time one hundred and fourteen children died in that vicinity with diphteria in one day,...*	He also told me, *that previous to that time the mortality had been very great among the children in that vicinity from diphtheria. As I now remember it 114 died in one day in the state,...*

1.1.23. Chapter XXIII

Page (1908)	1897	1908
273	...is a *liar.* ... drinks *his whiskey.*	... is a *falsifier.* ... drinks *whiskey for relief.*
273	She will be courting the moon that revolves around it, *without a living human soul on it,* in a few thousand years.	She will be courting the moon that revolves around it, in a few thousand years.
273	*Dr. Smith,...* . This *Dr. Smith* is our professor...	*Doctor,...* . This is our professor...
274	*Dr. Smith*: "I am not of that school now*, doctor; I am of your school".*	*He answered:* "I am not of that school now. *I see differently."*
276	I expect when I am gone that I will come back every week to see what Osteopathy is doing.	I expect when I am gone *from active earth life* that I will come back every week to see what Osteopathy is doing.
277	Specimens of what? *Of* the thoughtless stupidity of man, who, by taking medicine, has converted the *liver into a bank of cinnabar.*	Specimens of what? *Stones. Proof* of the thoughtless stupidity of man, who, by taking medicine, has converted the *organs into lime producers.*
278	*Dr. Smith...*	*Our anatomist...*
280	After you have *mastered* it...	After you have *studied it awhile...*
282	They *have* studied anatomy and physiology *to completion;...* and if they get puzzled, they come and ask me, and I *go to guessing.*	They studied anatomy and physiology;... and if they get puzzled, they come and ask me, and *I point them to the road leading to the cause.*
282	When you come here, you go in *there...*	When you come here, you go in *to the Infirmary...*
282	...and has spent twenty years in doing it, if he has failed to impart *that* knowledge he should quit.	...and has spent twenty *odd* years in doing it, if he has failed to impart *sufficient* knowledge *to others* he should quit.
283	...to demonstrate to you that I can teach it, and that men do know it.	...to demonstrate to you that I can teach it, and that men *can learn it and* do know it.
283	I know you can have it done *better* here.	I know you can have it done here.
285	*Dr. Smith,...*	*Doctor,...*

1.1.24. Chapter XXIV

Page (1908)	1897	1908
290	...thereby *set* the machinery of life *moving*. .... an attempt to *prove* God made a failure...	...thereby *restore* the machinery of life *to its normal movement*. ... an attempt to *show* God made a failure...
291	...by simply adjusting the vocal *organs. Deity created the organs, and also the law of their adjustment when out of order; neither did He mistake in the creation,* nor in the law. ... produced by *the use of calomel alone*.	...by simply adjusting the vocal *structure. Nature formed the organs, and framed the law of their adjustment and made no mistake in the formation*, nor in the law. . produced by *them.*
292-294		A LETTER TO MRS. ENQUIRER FROM MRS. EXPERIENCE.<br>Dear Friend, I send this missive forth the news to bear to thee,<br>That I, in thought, have wedded been to Osteopathy.<br>Now do not raise your hands and say, "She knoweth not her mind."<br>When you have heard my story all, your judgment will be kind.<br>In years agone when burning pain first seized me in its grasp,<br>I felt the need of a strong arm to help unloose its clasp.<br>The science (?) of Allopathy made promises most sweet;<br>I joined myself to it in heart —it seemed a partner meet—<br>The union most productive was of plasters, draughts and pills,<br>And did exceeding multiply my aches and pains and ills.<br>I found this match not heaven-made and if myself I'd save,<br>Must break the fetters of guess-work and so escape the grave.<br>Without the sanction of the law, myself I did divorce,<br>Joined hands with Homeopathy, which also proved a curse.<br>For little pills with sugar sweet and poisonous aconite,<br>Wrought greater mischief in my life than deadly dynamite;<br>From this new love I thought it best to quickly draw apart;<br>But where could I with safety find a place to rest my heart.<br>Hydropathy then forward stepped and unto me made suit,<br>This marriage quite prolific was in very watery fruit;<br>I soaked in baths of water cold and water on the boil,<br>Until my very joints did creak for want of needful oil.<br>Next, Hygiene I did espouse, its fruit and graham bread,<br>Its Swedish movements by machine, 'till I was almost dead.<br>It's mandate stern, a taste of salt in food would not allow.<br>Until the very tears I shed were fresh, I truly vow.<br>Again divorce came to my aid, but still I was in grief.<br>For all the helpmates which I sought had failed to give relief.<br>While deeply plunged in pain and woe, there was borne unto me,<br>The fame of a new Science, Grand, Fair Osteopathy.<br>This Science doth no offers make; it must be sought and won,<br>I tendered it my heart and hand, the thing was quickly done,<br>And now we stand united firm for all the coming years<br>To bear the fruit of health and love and banish pain and fears.<br>A locomotive Engineer each Osteopath cloth stand,<br>And guides his engine, mortal man, with true, unerring hand;<br>With master touch he doth adjust this engine's every part

		Nerves, muscles, bones and ligaments and e'en the throbbing heart<br>This science is exact and in accordance with its law<br>Each organ is revivified and acts without a flaw.<br>It gives man flesh, strength and health; makes him renew his youth<br>This grand result it doth attain without a drug, for- sooth.<br>So unto this, my new found love, I true and staunch will be,<br>And never will I be divorced from Osteopathy<br>I am most sure this match hath been of origin divine,<br>And so I wait your wishes good— as ever I am thine,<br>And hope that you may truly seek the way that brought me joy.<br>Sweet harmony it will restore and peace without alloy.<br>I did not think so much to write when first I did commence.<br>But you'll excuse, for just this once, your friend – Experience.<br>TEDDIE<br>Written for "Journal of Osteopathy," 1894.

to sentences highlighted in yellow and/or pink). The annotated chapters are available in the Supplementary material.

1.1.25. Chapter XXV

Page (1908)	1897	1908
295	...than all the *sciences known to human sympathy* combined.	...than all the *known sciences* combined.
296	...above all *things else*.	...above all *other methods of healing*.
298	...and let *not the weary* one fall by the wayside.	...and let *no* one fall by the wayside.
299		"OSTEOPATHY."<br>J. S. LOVELL.<br>The shades of night were falling fast,<br>As through a western village passed<br>A youth, who bore, without a friend,<br>A banner, with this strange legend.<br>Osteopathy.<br>In happy homes, he saw the light<br>Of joy go out in darkest night;<br>Bereft of friends by death's embrace,<br>Deep sorrow lined upon each face.<br>Osteopathy.<br>"Could I but stay the hand of death,"<br>The youth exclaimed,—"the vital breath,<br>God-given, hath perished soon,<br>Before the life had reached its noon,"<br>Osteopathy.<br>"Try not the pass," the doctors said,<br>"Dark lowers the tempest overhead,<br>Of persecution's awful power,<br>T'will fall upon you every hour,"<br>Osteopathy.<br>Undaunted now, with courage bold,<br>The youth did everywhere unfold,<br>The banner that so loud proclaimed<br>The science he had fitly named<br>Osteopathy.<br>The reign of error, like tyrant's rule,<br>Intolerant, save of its favorite school,<br>Was challenged by this champion bold,<br>Who to a suffering world had told<br>Osteopathy.<br>Long had their victims filled the ground,

		While tolling bells with solemn sound,<br>Rang out the death march to the grave,<br>Of thousands they had failed to save.<br>Osteopathy.<br>All hail! thou boon to all mankind,<br>That heals the sick, restores the blind;<br>Welcome! the bright and cheering ray,<br>That shines to mark thy dawning day,<br>Osteopathy.<br>Far up the height it now has climbed,<br>Leaving all other schools behind;<br>It's knowledge soon the earth shall fill,<br>While round the earth will echo "STILL,"<br>Osteopathy.

1.1.26. Chapter XXVI

Page (1908)	1897	1908
301	...by the doctors.	... by *whiskey* doctors.
302	...or to refuse to meet them, *would be to plead the baby act.*	...or to refuse to meet them, *is not my wish.*
302	...to save *the angels of my fireside*!	...to save *the loved ones of my household*!
303	...neither did they forget their snake medicine.	...neither did they forget their *whiskey or* snake medicine.
305	...said to Mr Morse...	...*is said to have* said to Mr Morse...
305	...act as *manure*...	...act as *a fertilizer*...
305	...for in such case medicine is of no avail.	...for in such case medicine is of no avail. *The shut off must be removed.*
306	If you can *learn all of* Osteopathy in *four* years I will buy you a farm, and a wife to run it *and* boss you.	If you can *learn* Osteopathy in *three* years I will buy you a farm, and a wife to run it *who will* boss you.
306	I do not understand a preacher's business. I have not made a study of the Bible;...	I do not understand a preacher's business. I have not made a study of the Bible *for that purpose*;...
306	God has forgotten nothing, and we find a supply of uric acid *for destroying stone in bladder or gall stones.*	God has forgotten nothing, and we find a supply of uric acid *which will destroy stone in the urinary bladder. His law is equally trustworthy in the destruction of gall stones.*
307	...opposition, merely regarding it as a fertilizer *of my work by* a fine quality of ignorance. ... and not on the *almighty* dollar...	...opposition, merely regarding it as a fertilizer *or* a fine quality of ignorance *which acts as an incentive, increasing my courage and determination. . .* ... and not on the dollar...
307	This, the 22d of June, is the anniversary of the child Osteopathy, the child of which I am justly proud. And today, on its coming of age, I am happy *and welcome you gladly.*	This, the 22d of June, is the anniversary *of the birth* of the child Osteopathy, the child of which I am justly proud. And today, on its coming of age, I am happy *to welcome you here.*
307-311		KANSAS BABY.<br>Way out in wind-swept Kansas in eighteen seventy-four<br>A babe lay girt in swaddling clothes – of it you will hear more.<br>It as a tiny youngster, not great in breadth or length,<br>But was possessed of Titan soul and will of wondrous strength.<br>No cradle song of love was crooned above its tiny bed,<br>For 'twas a homeless little waif – no place to lay its head.<br>No father with an air of pride clasped it in close embrace,

No mother with soft touch of lips rained kisses on its face.
But chill winds of indifference blew coldly on its form,
And those who guessed its parentage had wish to do it harm.
But God had meant that child to live and so to it he drew
The kindly care of Genius and Inspiration true.
The small young thing they did adopt and took it to their home.
Thence from the shelter of their love it never sought to roam.
They placed it in the lap of Thought, it nursed from Wisdom's breast
In bright robes of Intelligence its winsome form was dressed.
It wore the sandals white of Truth, was crowned with Purity,
And grew, as swiftly passed the years, a youth most fair to see.
He passed the hours in solitude, all quiet and alone,
The only playthings which he sought were various bits of bone.
So when his foster-parents called him Osteopathy,
They chose a fitting cognomen, I'm sure you will agree.
A ghastly skull with grinning teeth—a gruesome sight to see
An ulna, radius, femur or spinal vertebrae,
A clavicle or scapula gave to him intense delight.
He pored in studious thought o'er them from morn to dewy night.
And when he found their every use in nature's wondrous plan,
He dived yet deeper into thought and studied living man.
Brains, tissues, nerves and arteries and then, without a shiver,
Passed boldly on to lungs and heart, to diaphragm and liver.
This human engine soon he learned so skillfully to guide,
In quick response unto his touch it seemingly took pride.
The wonders that he daily wrought were told throughout the land,
And brought to him in search of health a sorry looking band.
But all their troubles fled away beneath his healing touch–
The paralytic rose and walked, the cripple dropped his crutch.
The blind received anew their sight, the dumb spoke loud in praise,
Till lo, the whole wide questioning world stood silent in amaze.
Yet there were foes on every hand, their hearts with envy rife.
Who sought, as Herod did of old, to take a young child's life.
Now if you'll kindly lend your ears, (I'll give them back again)
I'll tell you a history of these most wicked men.
Long years ago dark Ignorance with Superstition wed,
A horde of strong unruly boys they very quickly bred.
Bold Allopath, and Homeopath and young Eclectic too–
And Hydropath with vapor bath and each appliance new,
Electropath and Vitapath and other paths galore,
Until to name them every one would tax my memory sore,
Each bore strong hatred in his heart to every other one,
And strove to compass his downfall, from rise to set of sun.
But now in seeming friendship fair, they do their force unite
Against young Osteopathy to wage a bitter fight,

		They call their brave battallions forth and with most fiery glance<br>Against their foe, who stands alone, they do at once advance.<br>Their cannon balls are quinine pills, their gunshot pellets small.<br>Their bayonets are surgeon's knives, their canteens full of gall.<br>Their war song is—"Thou shalt not cure, while we have power to kill<br>For if your wonderous work goes on who will our graveyard fill."<br>But sturdy Osteopathy with shield of knowledge bright.<br>Meets them alone upon the field and puts their ranks to flight.<br>Altho' they strive with might and main they gain no vantage point,<br>And beat a double quick retreat with noses out of joint.<br>The victor's brow with laurel most fittingly is crowned,<br>The while with praise of his great deeds the whole earth doth resound.<br>This youth, grown up to man's estate, to-night doth come of age<br>And makes his twenty-first year bow to you upon this stage.<br>And now I'm sure you'll drink his health, clink glasses while we cry<br>God speed this science in its work and let it live for aye.<br>Written for Osteopathy's twenty-first birthday.<br>– TEDDIE.

1.1.27. Chapter XXVII

Page (1908)	1897	1908
312	...*Deific* plan...	...*Divine* plan...
313	The central figure of the group, *Mother* Sun, illumines space with *her* effulgent rays, and lights the pathway of numerous children and grandchildren too. *She* is a matchless *mother*, and guides *her* children well; each one of them is polished to the highest point of *perfection known to skill*. ... in the grand plan which *the mother has* on constant exhibition.	The central figure of the group, *Father* Sun, illumines space with *his* effulgent rays, and lights the pathway of numerous children and grandchildren too. *He* is a matchless *father*, and guides *his* children well; each one of them is polished to the highest point of *perfection*. ... in the grand plan which *is* on constant exhibition.
313	Small Mercury dwells close unto *her mother's* side, as if *she* feared to wander away lest *she* be lost in fields of space. *She* is arrayed in robes of vivid white, without a spot to mar *her* purity.	Small Mercury dwells close unto *his father's* side, as if *he* feared to wander away lest *he* be lost in fields of space. *He* is arrayed in robes of vivid white, without a spot to mar *his* purity.
313	...to gladden her *Mother's* heart and help increase the starry progeny. The eldest child of all, *Mrs. Uranus*, ... of the old *grandmother*... *Her* family... I saw *the gay, vivacious Mrs.* Saturn, with *her* many rings. *She* smiled on... Moon, *and* shed the light...	...to gladden her *Father's* heart and help increase the starry progeny. The eldest child of all, *Uranus*, ... of the old *grandparent*... *His* family... I saw Saturn, with *his* many rings. *He* smiled on... Moon, that *shed* the light...
314	...of the *lady Sun*, and followed with unfaltering footsteps the line of march *she had laid* out for them. I saw the face of the dear *mother* shrouded by a veil of impenetrable mourning, as if *her* heart	...of the *Sun*, and followed with unfaltering footsteps the line of march *laid* out for them. I saw the face of the dear *parent* shrouded by a veil of impenetrable mourning, as if *the* heart were

	were grieved by some erring action of one of *her* beauteous family. ... and revealed *her* face... . *She sent* this message...	grieved by some erring action of one of *the* beauteous family. ... and revealed *a* face... . *Sending* this message...
317	...no illegitimate *could* be allowed to run at large in Missouri. But the *it* soon...	...no illegitimate *would* be allowed to run at large in Missouri. But the *child* soon...
319	...appointed by the ancients of *rest*, ...because they *contain* sensation, motion, nutrition, assimilation, *and body completed*. ...I will give you a *handkerchief*...	...appointed by the ancients of *old*, ...because they *conduct life*, sensation, motion, nutrition, assimilation, *in the body when complete*. ...I will give you a *specimen*...
320	...have obtained the object sought, *without which no explorer ever succeeds.*	...have obtained the object sought.
321	Thought is the action of the machinery of the upper *or third* story of life, ...	Thought is the action of the machinery of the upper story of life, ...

1.1.28. Chapter XXVIII

Page (1908)	1897	1908
322	At the *end* of thirty-five *years*...	At the *age* of thirty-five...
323	Such *questions* arose... I found *to my mind* that there was a great mistake in God's work or in man's conclusions, if drugs were not in absolute demand when he was sick. ... to provide for His man ...and *He was* not the mind...	Such *thoughts* arose... I found *according to my idea* that there was *either* a great mistake in God's work or in man's conclusions, if drugs were not in absolute demand *within his system* when he was sick. ... to provide for His man ...and *say that He had* not the mind...
323	I was *dead, dead*, and folded in it to begin the common rest of all *human forms*, ...	I was *dead*, and folded in it to begin the common rest of all *humanity*, ...
324	As defined by Dunglison, "Surgery is that part of the healing art which relates to external diseases, their treatment, and especially to the manual operations adapted to their cure. "	As defined by Dunglison, "Surgery is that part of the healing art which relates to *injuries and* external diseases, their treatment, and especially to the manual operations adapted to their cure. "
326	What can you give us in place of drugs? we cannot add or give anything from the material world...	What can you give us in place of drugs? *we can give you adjustment of structure but* we cannot add or give anything from the material world...
326	... substances that have been made so by wear and motion.	... substances that have been made so by wear and motion.<br>*A perfectly adjusted body which will produce pure blood and plenty of it, deliver it on time and in quantity sufficient to supply all demands in the economy of life. This is what the osteopath can give you in the place of drugs if he knows his business.*

1.1.29. Chapter XXIX

Page (1908)	1897	1908
329	One more *circle* is added to that number...	One more *year* is added to that number...

330	He is surprised to find that man *is made by the eternal,* unerring Architect.	He is surprised to find that man *was made by an* unerring Architect.
330	The *thoughts of God himself are* found in every drop of your blood.	The *wisdom of Nature's architect is* found in every drop of your blood.
332	...from the bosom of *God.*	...from the bosom of *Nature.*
332	She is hungry for a longer lease on that child's *days.*	She is hungry for a longer lease on that child's *life.*
334	*The tally-sheet says No.* Look the world over....	Look the world over...

1.1.30. Chapter XXX

Page (1908)	1897	1908
336-337		THE RHYME OF THE BONES.<br>How many hones in the human face?<br>Fourteen, when they are all in place,<br>How many bones in the human head?<br>Eight, my child, as I've often said.<br>How many bones in the human ear?<br>Three in each, and they help to hear.<br>How many bones in the human spine?<br>Twenty-six, like a climbing vine.<br>How many bones in the human chest?<br>Twenty-four ribs, and two of the rest.<br>How many bones the shoulders bind?<br>Two in each; one before, one behind.<br>How many bones in the human arm?<br>In each arm one, two in each forearm.<br>How many bones in the human wrist?<br>Eight in each if none are missed.<br>How many bones in the palm of the hand?<br>Five in each. with manv a band.<br>How many bones in the fingers ten?<br>Twenty-eight, and by joints they bend.<br>How many bones in the human hip?<br>One in each, like a dish they dip.<br>How many bones in the human thigh?<br>One in each, and deep they lie.<br>How many bones in the legs at the knees?<br>One in each, the knee pan, please.<br>How many bones in the leg from the knee?<br>Two in each – we can plainly see.<br>How many bones in the ankle strong?<br>Seven in each, but none are long.<br>How many bones in the ball of the foot?<br>Five in each, as in the palms were put.<br>How many bones in the toes half-a-score?<br>Twenty-eight, and there are no more.<br>And now, all together these many bones fix,<br>And they count in the body two hundred and six.<br>And then we have in the human mouth,<br>Of upper and under, thirty-two teeth.<br>And we now and then have a bone I should think,<br>That forms on a joint, or fills up a chink.<br>A Sesamoid bone, or a wormian we call.<br>And now we may rest, for we've told them all.
339	...the blood of brothers was spilled in this city by the cannon, sword *and cold lead.*	...the blood of brothers was spilled in this city by the cannon, *and* sword.
340	This is My medical library, surgery, and obstetrics. This is My book with all directions, instructions,	This is My medical library, *My* surgery, and *My* obstetrics. This is My book with all *the* directions,

	doses, sizes, and quantities to be used in *all cases* of sickness, and birth, the beginning of man; in childhood, youth, and declining days." I am *something of* what ...	instructions, doses, sizes, and quantities to be used in *every case* of sickness, and birth, the beginning of man; in childhood, youth, and declining days." I am *some times* what ...
340	... and you should become sick*, which makes poisons in the blood, stagnation, making a new matter* in it, ...	... and you should become sick*, and poison generated in the blood, stagnation forming a new substance* in it, ...
340	I commenced the hunt when but a child, and kept it up *until I was forty years old.*	I commenced the hunt when but a child, and kept it up, *and am still on the hunt.*
341	*They* have a system of naming them; we call it symptomatology. You put the parts all together, and you have made a something, and *that is* croup.	*We* have a system of naming them; we call it symptomatology, *which is an arbitrary system of naming things, whether comprehended or not.* You put the parts all together, and you have made a something, and *you call it* croup.
342	The wise Architect of the universe put that mill *there* and ... when He *put them there.*	The wise Architect of the universe put that mill *within you* and ... when He *planned them.*
343	The arteries bring the blood *and wash it with the spirit of life.*	The arteries bring the blood *of life and construct man, beast and all other bodies.*

1.1.31. Chapter XXXI

Page (1908)	1897	1908
353	As I had no money I could purchase no more and had to travel many tiresome miles alone. ... superior gifts, only success.	As I had no money I could purchase no more *budgets of success* and had to travel many tiresome miles alone. ... superior gifts, only success *in every effort.*

1.1.32. Chapter XXXII

Page (1908)	1897	1908
366	*Through the kindness of Funk & Wagnalls Company, of New York, we are permitted this elaborate cut, which shows about one-fourth of the muscles of the human body, each of which is a useful servant in performing the labors of life. I give place to those beautiful pictures of some of the parts of that greatest of all known machines, who bears the name of man.*	[First paragraph and table of muscles have been cut]
367	...but a few thoughts given to this field of philosophy, *with a few illustrations*, may cause you...	...but a few thoughts given to this field of philosophy, may cause you...
369	...to *Dr Smith's* room for an autopsy.	...to *Dr Jones's* room for an autopsy.
370	Off to the country I went with a flour-sack full of darkey bones in 1877. and have been *doing so* ever since.	Off to the country I went with a flour-sack full of darkey bones in 1877. and have been *studying bones* ever since.
373	...William *Novenger*,... ... Captain *Bumpass.*	...William *Novinger*,... ... Captain *Bumpus.*

1.1.33. Chapter XXXIII

Page (1908)	1897	1908
381	... which is *“No,” all day.* We ...	... which is “*No.” All day.* We ...
385		LINES ON A SKELETON.<br>(The following is a poem found near a human skeleton in the Royal College of Surgeons of London, and a reward of fifty guineas failed to discover its author.)<br>"Behold this ruin! 'Twas a skull<br>Once of ethereal spirit full.<br>This narrow cell was life's retreat;<br>This space was thought's mysterious seat.<br>What beauteous visions filled this spot!<br>What dreams of pleasure long forgot!<br>Nor hope, nor joy, nor love, nor fear,<br>Has left one trace of record here.<br>Beneath this mouldering canopy<br>Once shone the bright and busy eye.<br>But start not at the dismal void;<br>If social love that eye employed;<br>If with no lawless fire it gleamed;<br>But thro' the dews of kindness beamed-<br>That eye shall be forever bright.<br>When stars and suns are sunk in night.<br>"Within this hollow cavern hung,<br>The ready, swift and tuneful tongue.<br>If falsehood's honey is disdained.<br>And when it could not praise, was chained;<br>If bold in virtue's cause it spoke,<br>Yet gentle concord never broke-<br>This silent tongue shall plead for thee,<br>When Time unveils Eternitv!<br>"Say. did these fingers delve the mine,<br>Or with its envied rubies shine?<br>To hew the rock or wear the gem,<br>Can nothing now avail to them;<br>But if the page of truth they sought,<br>Or comfort to the mourner brought-<br>These hands a richer mead shall claim<br>Than all who waits on wealth or fame.<br>"Avails it whether bare or shod,<br>These feet the path of duty trod?<br>If from the bowers of ease they fled,<br>To seek affliction's humble shed;<br>If grandeur's guilty bribe they spurned<br>And home to virtue's cot returned<br>These feet with angels wings shall rise,<br>And tread the palace of the skies.

1.1.34. Additional pages in the 1908 version

Page (1908)	1897	1908

387-<br>390		The past ten years in osteopathy<br>(by Franklin Fiske, A.B. D.O.)<br>p.388 William Smith is mentioned as faculty member
390-<br>91		Contribution by J.A. Quintal, D.O.
392-<br>396		Four noted cranks (a story in four parts)<br>By a Friend
397-<br>399		Some early history of Doctor Still<br>By A.A.B. Caveness
400-<br>401		Conclusion by A.T. Still
402-<br>403		Osteopathy – Our calling<br>By A.S.J. Lehr, St. Louis, Mo.
